# A Buffered LC‐MS Method for Resolving and Quantifying Albiflorin and Paeoniflorin

**DOI:** 10.1002/bmc.70353

**Published:** 2026-01-18

**Authors:** Alina Gazizova, Ela Hümay Altincubuk, Christina Oppermann

**Affiliations:** ^1^ Industrial and Analytical Chemistry, Department of Chemistry University of Rostock Rostock Germany

## Abstract

Albiflorin and paeoniflorin are bioactive isomers found in *Paeoniae Radix Alba*, a key component of Traditional Chinese Medicine. Their accurate and simultaneous quantification is essential for pharmacological research. Standard separation methods often rely on LC‐MS using a mobile phase containing 0.1% formic acid. This study demonstrates that the use of 0.1% formic acid generates a secondary peak for albiflorin that exhibits an identical mass‐to‐charge ratio and similar fragmentation pattern as the main peak. To address this, an LC‐MS method was developed using a Kinetex phenyl‐hexyl column with a gradient elution using a buffered mobile phase of 10 mM ammonium acetate and acetic acid (pH 4.4) in water and methanol. The method was validated for linearity, precision, accuracy, and sensitivity. It successfully separated paeoniflorin and albiflorin preventing the formation of the albiflorin artifact. The method demonstrated good linearity over a concentration range of 1–50 μg/mL. Applicability was tested through the analysis of a *Paeoniae Radix Alba* extract. The developed LC‐MS method enables accurate and simultaneous quantification of albiflorin and paeoniflorin by eliminating the formation of a second albiflorin peak. This makes the method potentially suitable for pharmacological studies of *Paeoniae Radix Alba* and other plants containing albiflorin and paeoniflorin.

**Abbreviations**
arb: arbitrary
aux: auxiliary
ESI: electrospray ionization
LC: liquid chromatography
MS: mass spectrometry
NMR: nuclear magnetic resonance

## 1 Introduction

Albiflorin and paeoniflorin are monoterpene glucosides and can be found in the extract of *Paeoniae Radix Alba*, the root of *Paeoniae lactiflora*, which is commonly used in Traditional Chinese Medicine. Pharmacological research has shown that both albiflorin and paeoniflorin possess important health benefits. Albiflorin has demonstrated anti‐inflammatory (Wang et al. 2014), antioxidative (Fang et al. 2023; Ho et al. 2015), neuroprotective (Fang et al. 2023; Ho et al. 2015; Ma et al. 2021; Park et al. 2025; Xu et al. 2019), and hepatoprotective properties (Liu et al. 2024; Song et al. 2024). Paeoniflorin also exhibits anti‐inflammatory (Wang et al. 2014) and antioxidative effects (He and Dai 2011). In addition, it also possesses immunomodulatory (He and Dai 2011) and hematopoietic (Zhu et al. 2016) properties. The reliable and accurate quantification of these compounds is essential for their pharmacological research.

Liquid chromatography (LC) coupled with mass spectrometry (MS) is the preferred method for the analysis of complex mixtures such as plant extracts due to its high sensitivity and specificity. Albiflorin and paeoniflorin are structural isomers, which makes their chromatographic separation challenging. Established methods for separating these isomers predominantly utilize reversed‐phase (C18) columns and with a mobile phase consisting of water and an organic solvent (acetonitrile) acidified with 0.1% formic acid (Sheng et al. 2004; Tong et al. 2010). However, during our attempts to replicate these procedures, the formation of a minor second peak in the albiflorin chromatogram was observed. It eluted several minutes after the main peak and exhibited similar MS and MS^2^ spectra. After thorough investigation, we found that the presence of 0.1% formic acid consistently led to minor peak formation. Complete removal of the 0.1% formic acid, however, resulted in a loss of chromatographic resolution between the two isomers. Therefore, we explored the implementation of a buffered mobile phase. A publication by Jeon et al. utilized an eluent system consisting of acetonitrile and 5 mM sodium phosphate buffer at pH 3.0 for the separation of albiflorin and paeoniflorin (Jeon et al. 2009). However, phosphate buffers are not suitable for LC‐MS due to their low volatility (Dolan 2006; Shimadzu Deutschland GmbH, n.d.). More recently, Bao et al. described an LC‐MS method using a volatile ammonium acetate buffer in combination with 0.1% formic acid for the quantitative analysis of paeoniflorin alone. This method yielded good results; however, the separation of albiflorin and paeoniflorin was not investigated in this study (Bao et al. 2022). Addressing this gap, we developed a method using a mobile phase consisting of water with 10 mM ammonium acetate and acetic acid (pH 4.4) and methanol. This approach resolves albiflorin and paeoniflorin while preventing the formation of the acid‐induced albiflorin artifact, allowing for accurate and simultaneous quantification.

## 2 Experimental

### 2.1 Chemicals and Plant Material

Solvents for LC‐MS analysis (LC‐MS quality) were obtained from Fisher Chemicals (Fisher Scientific GmbH, Schwerte, Germany). Ammonium acetate (Optima, LC‐MS) and glacial acetic acid (laboratory reagent grade) were also purchased from Fisher Chemicals. Formic acid (99%–100%) was bought from VWR Chemicals (VWR International GmbH, Darmstadt, Germany).

Deuterated methanol and deuterated dimethylsulfoxide were bought from Sigma‐Aldrich (Sigma‐Aldrich Chemie GmbH, Schnelldorf, Germany).

Paeoniflorin and albiflorin standards (both >98%) were purchased from Cayman Chemicals (Ann Arbor, Michigan, USA) and an additional Albiflorin standard (>97%) was bought from PhytoLab GmbH & Co. KG (Vestenbergsgreuth, Germany). Both standards were dissolved in ethanol (≥99.5%, Sigma‐Aldrich Chemie GmbH, Schnelldorf, Germany).

*Paeoniae Radix Alba* (Bai Shao Yao) was provided by Chinasan GmbH & Co. KG (Aachen, Germany). Methanol (HPLC grade) used for extraction was bought from Fisher Chemicals (Fisher Scientific GmbH, Schwerte, Germany).

### 2.2 Instruments and Analysis Methods

LC‐MS analysis was performed using an UltiMate 3000 liquid chromatograph coupled with an LTQ XL mass spectrometer (both obtained from Thermo Fisher Scientific, Schwerte, Germany). For separation, a Kinetex phenyl‐hexyl (150 × 2.1 mm × 2.6 μm) column (Phenomenex, Aschaffenburg, Germany) was used and the eluent consisted of methanol (A) and water (B) both with an addition of 0.1% formic acid in the beginning and was changed to methanol (A) and water buffered with 10 mM ammonium acetate and glacial acetic acid at pH 4.4 (B). The pH was adjusted using a SevenCompact Duo pH/Conductivity Meter (Mettler Toledo Inc., Columbus, OH, USA). In both cases, the following gradient was used at a flow rate of 250 μL/min: 0–1 min 95% B, 1–2 min 95%–60% B, 2–7 min 60% B, 7–8 min 60%–40% B, 8–15 min 40% B, 15–20 min 40%–95% B, 20–25 min 95% B. The column temperature was set to 45°C and the injection volume was 3 μL. Ionization was performed using electrospray ionization (ESI) with the following settings: *m*/*z* 50–2000, negative and positive scan mode, sweep gas flow rate: 10 arb. units, aux. gas flow: 5 arb. units, sheath gas flow: 35 arb. units, spray voltage: −3.0 kV, capillary temperature: 275°C, capillary voltage: −30.0 V, tube lens voltage: 30 V, H‐ESI source heater: 300°C).

### 2.3 Preparation of Calibration Standards and Quality Control Samples

A paeoniflorin stem solution was prepared by dissolving the standard in ethanol to yield a concentration of 48.0538 mg/mL (100 mM). For the albiflorin stem solution, 1 mg of the sample was weighed on an analytical balance (VWR, Electronic Precision balance SM, VWR International GmbH, Darmstadt, Germany) and dissolved in ethanol to a final concentration of 1 mg/mL.

### 2.4 Plant Material Preparation and Extraction

*Paeoniae Radix Alba* was ground using a coffee mill (Bosch TSM6A013B, Robert Bosch GmbH, Gerlingen‐Schillerhöhe, Germany). The resulting powder was extracted using a CEM Mars XPress (Kamp‐Lintfort, Germany) laboratory microwave by extracting 12.0771 g powder with 108 mL methanol using the following program: 60°C, 400 MW (5 min warming, 10 min constant heating, 5 min cooling). After extraction, the liquid phase was carefully decanted and filtered using a Millipore system with a Durapore (both Merck KGaA, Darmstadt, Germany) filter (pore size 0.22 μm). The powder was extracted with fresh methanol a total of four times. The solvent was removed in vacuo, yielding 1.4082 g extract.

A 0.025‐mg sample of the resulting extract was taken for LC‐MS measurements and dissolved in 1 mL methanol. The sample was measured a total of three times.

### 2.5 Method Validation

#### 2.5.1 Selectivity

The selectivity of the method was demonstrated by comparing the chromatograms of an ethanol blank and a sample spiked with albiflorin and paeoniflorin. The samples were integrated and compared to chromatograms of the lowest calibration standard.

#### 2.5.2 Limit of Detection and Limit of Quantification

Calibration curves in ethanol at seven concentration levels (0.001, 0.002, 0.003, 0.005, 0.010, 0.025, and 0.05 mg/mL) were prepared and measured a total of five times to assess the linearity of the method. The limits of detection (LOD) and quantification (LOQ) were determined from the equations of the calibration curves as follows:

$$\mathrm{LOD}=3\;\frac{\sigma }{s}$$

$$\mathrm{LOQ}=10\;\frac{\sigma }{s}$$

where *σ* is the error of the interception with the *y*‐axis and *s* is the slope of the calibration curve.

#### 2.5.3 Precision and Accuracy

Precision and accuracy were evaluated by preparing samples with different concentrations of albiflorin and paeoniflorin (Table 1) and measuring them a total of five times. Determined concentrations were compared to expected concentrations of the samples.

**TABLE 1:** Nominal concentrations of albiflorin and paeoniflorin in six validation samples used for method validation.

Sample No.	*c*_albiflorin_ (mg/mL)	*c*_paeoniflorin_ (mg/mL)
1	0.00200	0.03500
2	0.03800	0.00220
3	0.00520	0.00490
4	0.00856	0.02520
5	0.02740	0.00826
6	0.00932	0.00947

### 2.6 NMR Analysis

For analysis via nuclear magnetic resonance (NMR), two samples were prepared containing approximately 4.5 mg of the albiflorin standard, which was dissolved in approximately 0.7 mL DMSO‐d6 and MeOD‐d4. Measurements were performed on a 500 MHz device (Bruker AVANCE 500 Neo). The measurements were repeated after the addition of 1 and 10 μL formic acid.

### 2.7 Software

LC‐MS measurements were performed and processed using XCalibur (version 2.0.0.0, Thermo Fisher Scientific, Schwerte, Germany). Graphs were prepared using Origin Pro 2025 (OriginLab Corporation, Northampton, MA, USA). NMR spectra were analyzed using TopSpin (version 4.5.0., Bruker).

## 3 Results and Discussion

### 3.1 Method Development

#### 3.1.1 Analysis Method With Formic Acid

**FIGURE 1:**
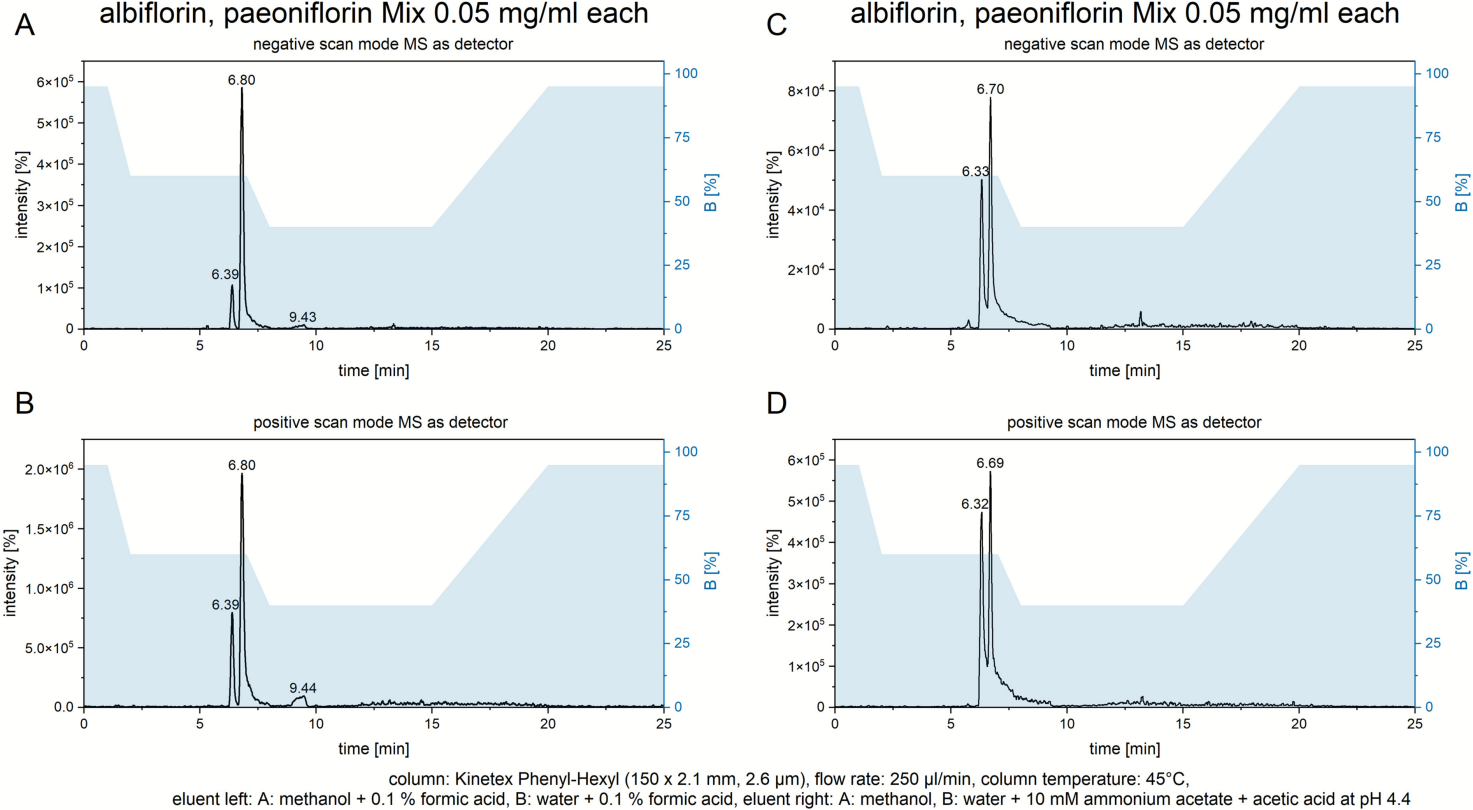
Chromatograms of 0.05 mg/mL mixtures of albiflorin and paeoniflorin measured on a Kinetex phenyl‐hexyl column using an eluent system containing formic acid in negative (A) and positive (B) scan modes and an eluent system containing pure methanol and water with 10 mM ammonium acetate and acetic acid buffered at pH 4.4 in negative (C) and positive (D) scan modes.

At first, a method using an eluent consisting of methanol and water both with the addition of 0.1% formic acid was used to measure 0.1 mg/mL standards of both analytes separately, as well as a mixture of both analytes at 0.05 mg/mL. Exemplary chromatograms of the mixture in the positive and negative scan modes are shown in Figure 1A,B

The retention times of albiflorin and paeoniflorin were determined from the measurements of pure standards. The chromatogram of paeoniflorin shows a singular peak at 6.80 min; however, the chromatogram of albiflorin displays two peaks, one main peak at 6.39 min and a broader and less intensive secondary peak at 9.44 min. Mass spectra of both peaks in the albiflorin chromatogram were nearly identical (Supplementary Figure 1).

#### 3.1.2 Analysis Method With 10 mM Ammonium Acetate and Acetic Acid at pH 4.4

The influence of different parameters such as column material, column temperature, eluent composition, and gradient on the chromatogram of albiflorin were tested. It was found that the second peak was present after separation on two different columns and using an eluent gradient of acetonitrile and water, each with an addition of 0.1% formic acid. Isocratic elution with methanol and water, each with an addition of 0.1% formic acid, also showed the second peak in the albiflorin chromatogram. Furthermore, lower column temperatures (40°C, 35°C, and 30°C) also had no effect on the second peak.

Since the second signal in the chromatogram of the albiflorin standard was not visible with an eluent containing 10 mM ammonium acetate and acetic acid buffered at a pH of 4.4, a method using this eluent was developed. Chromatograms of a mixture of albiflorin and paeoniflorin at 0.05 mg/mL each measured with this method are displayed in Figure 1 C and D. The peaks were assigned by measuring pure standards separately.

The retention time of albiflorin shifts only slightly from 6.39 to 6.32 min, while the retention time of paeoniflorin undergoes a stronger shift from 6.80 to 6.70 min with the buffered eluent.

### 3.2 Method Validation

#### 3.2.1 Selectivity

The selectivity was determined in accordance with ICH guideline M10 (European Medicines Agency 2022) by processing the chromatograms of five blank (ethanol) sample measurements with the lowest calibration sample containing both substances at a concentration of 0.001 mg/mL.

Even at the lowest concentrations tested, the standards are detected with peak areas of 9688 ± 1261.66 (negative) and 111,808 ± 9003.68 (positive) for albiflorin and 25,810 ± 2662.7736 (negative) and 93,984 ± 2680.7669 (positive) for paeoniflorin, while no signal is found in the ethanol blanks. This shows sufficient selectivity of the method.

#### 3.2.2 Limit of Detection and Limit of Quantification

Calibration standards of albiflorin and paeoniflorin were prepared and analyzed in five replicate injections. The resulting chromatograms were integrated using a processing method in XCalibur.

Determined detection limits (LOD) and quantification limits (LOQ) are shown in Table 2.

**TABLE 2:** Limit of detection (LOD) and limit of quantification (LOQ) for albiflorin and paeoniflorin in negative and positive scan modes.

	Negative scan mode		Positive scan mode	
Compound	LOD (mg/mL)	LOQ (mg/mL)	LOD (mg/mL)	LOQ (mg/mL)
Albiflorin	0.00148	0.00448	0.00256	0.00775
Paeoniflorin	0.00114	0.00347	0.00259	0.00786

Although signal intensities were generally higher in positive scan mode, the LOD and LOQ values of analytes were consistently lower in negative scan mode. This can be attributed to increased background noise or possible matrix‐induced ion suppression, which reduces signal‐to‐noise (S/N) ratios and impairs detection limits (Lupo and Kahler 2017). Overall, negative scan mode demonstrated better sensitivity and is therefore more suitable for quantitative analysis of albiflorin and paeoniflorin.

#### 3.2.3 Precision and Accuracy

Samples with varying concentrations of albiflorin and paeoniflorin were according to the nominal concentrations in Table 1. They were analyzed alongside the calibration standards and integrated using the same processing method. Each sample was measured five times. Means and standard deviations were calculated and compared to the nominal concentrations. The results are summarized in Table 3.

**TABLE 3:** Measured concentrations, recovery rates and error percentages for albiflorin and paeoniflorin in negative and positive scan modes.

Negative scan mode								
Sample No.	Determined *c*_albiflorin_ (mean ± standard deviation) (mg/mL)	Relative standard deviation (%)	Recovery (%)	Recovery error (%)	Determined *c*_paeoniflorin_ (mean ± standard deviation (mg/mL)	Relative standard deviation (%)	Recovery (%)	Recovery error (%)
1	0.00252 ± 0.00051	20.16	125.83	25.83	0.03328 ± 0.00253	7.59	95.09	−4.91
2	0.03745 ± 0.00135	3.60	98.55	−1.45	0.00230 ± 0.00032	13.83	104.70	4.70
3	0.00508 ± 0.00016	3.18	97.61	−2.39	0.00526 ± 0.00023	4.41	107.44	7.44
4	0.00944 ± 0.00131	13.91	110.30	10.30	0.02653 ± 0.00150	5.66	105.26	5.26
5	0.02658 ± 0.00126	4.74	97.02	−2.98	0.00904 ± 0.00046	5.07	109.48	9.48
6	0.00952 ± 0.00020	2.08	102.17	2.17	0.00978 ± 0.00034	3.44	103.25	3.25

Positive Scan mode								
Sample No.	Determined *c*_albiflorin_ (mean ± standard deviation) (mg/mL)	Relative standard deviation (%)	Recovery (%)	Recovery error (%)	Determined *c*_paeoniflorin_ (mean ± standard deviation (mg/mL)	Relative standard deviation (%)	Recovery (%)	Recovery error (%)
1	0.00186 ± 0.00093	50.23	92.80	−7.20	0.03058 ± 0.00254	8.30	87.38	−12.62
2	0.03249 ± 0.00161	4.96	85.51	−14.49	0.00283 ± 0.00133	47.01	128.74	28.74
3	0.00441 ± 0.00042	9.51	84.73	−15.27	0.00409 ± 0.00031	7.65	83.49	−16.51
4	0.00850 ± 0.00060	7.01	99.30	−0.70	0.01862 ± 0.00240	12.87	73.88	−26.12
5	0.02413 ± 0.00098	4.06	88.07	−11.93	0.00792 ± 0.00049	6.12	95.93	−4.07
6	0.00873 ± 0.00019	2.19	93.69	−6.31	0.00856 ± 0.00087	10.10	91.30	−8.70

In negative scan mode, recoveries ranged from 97.02% to 125.83% for albiflorin and 95.09% to 109.48% for paeoniflorin. All values were within the generally accepted accuracy limits of ±15% (European Medicines Agency 2022), except for the albiflorin concentration in sample 1, which was overestimated by +25.83%. This deviation is expected, as the nominal concentration (0.00200 mg/mL) of this sample was above the LOD but below the LOQ, where accurate quantification is not guaranteed. The precision in negative scan mode was generally satisfactory with %RSD < 15% for nearly all samples, except for the albiflorin concentrations in sample 1 (20.16%), which can also be explained by the low nominal concentration in the sample.

In positive scan mode, recoveries varied stronger (84.73%–99.30% for albiflorin and 83.49%–128.74% for paeoniflorin). Precision values were outside of acceptable range for the albiflorin concentration in sample 1 and the paeoniflorin concentration in sample 2 (50.23% and 47.01%, respectively). In both cases, the nominal analyte concentrations in the samples (0.00200 mg/mL for albiflorin and 0.00220 mg/mL for paeoniflorin) were below LOD (0.00256 mg/mL for albiflorin, 0.00259 for paeoniflorin), where not only reliable quantification but also dependable detection is not expected. At concentrations above the LOQ, however, %RSD values generally fell below 10%, indicating reliable repeatability at higher concentrations.

Overall, the negative scan mode provided superior accuracy and precision across the concentration range, while the positive scan mode showed lower accuracy, likely due to matrix effects and ion suppression (Cech and Enke 2001).

### 3.3 Determination of Albiflorin and Paeoniflorin Concentrations in *Paeoniae Radix Alba* Extract

The developed method was applied to quantify albiflorin and paeoniflorin in a methanolic microwave extract of *Paeoniae Radix Alba*. As measurements in negative scan mode demonstrated better selectivity and accuracy during validation, only results obtained in this mode are reported. The results are demonstrated in Table 4.

**TABLE 4:** Albiflorin and paeoniflorin concentrations determined in *Paeoniae Radix Alba extract in negative scan mode*.

Sample No.	*c*_albiflorin_ (mg/mL)	*c*_paeoniflorin_ (mg/mL)
1	0.00162	0.00628
2	0.00155	0.00568
3	0.00170	0.00624
Mean ± standard deviation	0.00162 ± 0.00008	0.00607 ± 0.00034

The determined albiflorin concentration was above LOD but below LOQ, permitting only semiquantitative assessment. For paeoniflorin, the measured concentration was above the LOQ, allowing reliable quantification.

Based on the measured concentrations in 0.025 mg extract dissolved in 1 mL ethanol, the amount of albiflorin and paeoniflorin in the total extract (1.4082 g) were calculated to be 64.81 ± 3.04 mg/g_extract_ and 242.61 ± 13.53 mg/g_extract_, respectively. This corresponds to 7.56 ± 0.35 mg/g_powder_ albiflorin and 28.29 ± 1.58 mg/g_powder_ paeoniflorin in the crude *Paeoniae Radix Alba* powder. These results are consistent with albiflorin and paeoniflorin contents previously reported in the literature for *Paeoniae Radix Alba* (Gao et al. 2015; Tan et al. 2020; Zhang et al. 2022), confirming both the reliability of the method and its applicability to phytochemical analysis.

### 3.4 NMR Analysis

**FIGURE 2:**
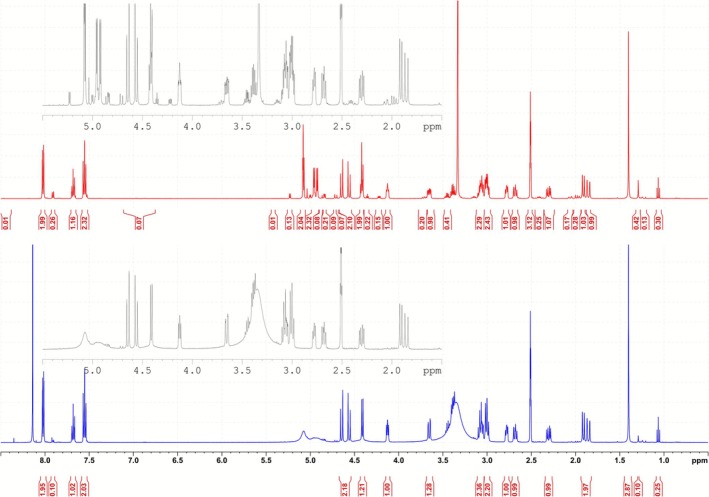
^1^H NMR spectrum of albiflorin before (top, red) and after (bottom, blue) addition of formic acid measured in deuterated dimethylsulfoxide.

To study the effect of acid on the albiflorin standard, a sample was dissolved in deuterated dimethylsulfoxide and deuterated methanol and measured before and after the addition of formic acid. The ^1^H and ^13^C NMR spectra are shown in Figure 2 and 2D‐NMR spectra can be viewed in the Supporting Information. In the ^1^H NMR spectrum, two species can be seen, even before the addition of acid. The signals of the main species can be clearly identified as belonging to albiflorin in comparison with the literature (Shi et al. 2016). Peaks of the second species are much less intensive, with an approximate ratio of 1:10 of subspecies to main species.

The NMR spectra before and after acid addition do not display any differences with the main species. Signals belonging to the second species do seem to display differences; however, it is present only in traces and the amount is not enough to study in ^13^C or 2D‐NMR.

## 4 Conclusion

In this study, we developed and validated an LC‐MS method for the simultaneous quantification of albiflorin and paeoniflorin. During method development, a secondary minor peak was observed in albiflorin chromatograms across different columns and eluent systems. Investigations indicated the addition of 0.1% formic acid as the likely cause of the second peak. After switching to an acid‐free eluent system, the minor peak could no longer be detected.

Extensive NMR studies were conducted to study the mechanism of formation of the minor peak. However, these investigations were inconclusive. The absence of additional major peaks or peak shifts suggested that the mechanism likely did not involve either isomerization or significant conformational changes.

The developed method employed a gradient elution with methanol and water containing 10 mM ammonium acetate, buffered to pH 4.4 with acetic acid. Method validation demonstrated sufficient sensitivity, precision, and accuracy. Despite lower signal intensities, negative ionization mode seemed to be better due to lower LOD and LOQ, as well as better accuracy across the tested concentration range.

Furthermore, the method was applied to *Paeoniae Radix Alba* extract and yielded an albiflorin level of 7.56 ± 0.35 mg/g_powder_ (semiquantitatively) and a paeoniflorin level of 28.29 ± 1.58 mg/g_powder_ (quantitatively), which is consistent with values reported in literature.

In the future, this method might be adapted for more complex matrices such as cell culture medium. Preliminary blank experiments indicated no significant matrix interference. However, for quantification, the method will require further validation due to potential matrix effects or possible ion suppression, particularly in the positive scan mode (Lupo and Kahler 2017).

Overall, the developed method is suitable for simultaneous quantification of albiflorin and paeoniflorin in herbal extracts and may be suitable for application in more complex matrices such as cell culture medium. However, additional validation would be required to ensure sensitivity and reproducibility under such conditions.

## Author Contributions

C.O. conceived the project. All authors contributed to the study conception and design. Material preparation, data collection, and analysis were performed by A.G. and E.A. The first draft of the manuscript was written by A.G., and all authors commented on previous versions of the manuscript. All authors read and approved the final manuscript.

## Funding

The LTQ XL LC‐MS system was cofinanced by the European Union (European Regional Development Fund GHS‐17‐0034 under the Operational Program Mecklenburg‐Vorpommern 2014–2020—Investments in growth and employment).

## Consent

The authors have nothing to report.

## Conflicts of Interest

The authors declare no conflicts of interest.

## Ethics and Consent to Participate

Herewith, all authors confirm that all methods were carried out in accordance with relevant guidelines and regulations.

## Declarations

Not relevant.

## Supporting information


**Data S1:** Supporting information.

**Figure S1:** Mass spectra of paeoniflorin and albiflorin standards in negative and positive scan mode. Spectra A–F were obtained using the nonbuffered eluent system and spectra G–J were measured using the buffered eluent system. A, B, G, and H show paeoniflorin, while C, D, I, and J show albiflorin. E and F display the mass spectrum of the second peak found in the chromatogram of the albiflorin standard measured with method 1.

**Figure S2:** bmc70353‐sup‐0003‐Figure_S2.png. ^13^C‐NMR spectrum of albiflorin standard before (top, red) and after (bottom, blue) formic acid addition.

**Figure S3:** DEPT‐NMR spectrum of albiflorin standard before (top, red) and after (bottom, blue) formic acid addition.

**Figure S4:** COSY‐NMR spectrum of albiflorin standard before (red) and after (blue) formic acid addition.

**Figure S5:** bmc70353‐sup‐0006‐Figure_S5.png. ^1^H‐^13^C‐HSQC spectrum of albiflorin standard before (red) and after (blue) formic acid addition.

**Figure S6:** bmc70353‐sup‐0007‐Figure_S6.png. ^1^H‐^13^C‐HMBC spectrum of albiflorin standard before (red) and after (blue) formic acid addition.

**Figure S7:** NOESY spectrum of albiflorin standard before (red) and after (blue) formic acid addition.

**Figure S8:** TOCSY spectrum of albiflorin standard before (red) and after (blue) formic acid addition.

**Figure S9:** bmc70353‐sup‐0010‐Figure_S9.png. ^1^H‐NMR spectrum of albiflorin after addition of 1 μL (top, red) and 10 μL (bottom, dark red) of formic acid.

**Figure S10:** 1H‐NMR spectrum of albiflorin before (top, red) and after (bottom, blue) addition of formic acid.

**Figure S11:** bmc70353‐sup‐0012‐Figure_S11.png. ^13^C‐NMR spectrum of albiflorin standard before (top, red) and after (bottom, blue) formic acid addition.

**Figure S12:** DEPT‐NMR spectrum of albiflorin standard before (top, red) and after (bottom, blue) formic acid addition.

**Figure S13:** COSY‐NMR spectrum of albiflorin standard after formic acid addition.

**Figure S14:** bmc70353‐sup‐0015‐Figure_S14.png. ^1^H‐^13^C‐HSQC spectrum of albiflorin standard after formic acid addition.

**Figure S15:** 1H‐13C‐HMBC spectrum of albiflorin standard after formic acid addition.

**Figure S16:** bmc70353‐sup‐0017‐Figure_S16.png. ^1^H‐NMR spectrum of albiflorin after addition of 1 μL (top, red) and 10 μL (bottom, dark red) of formic acid.

**Figure S17:** Calibration curves of albiflorin (A, B) and paeoniflorin (C, D) in negative and positive scan modes. The fitted calibration equations, coefficients of determination (*R* ^2^) and 95% confidence bands are shown.

**Figure S18:** Measured concentrations of albiflorin (blue) and paeoniflorin (green) in the six validation samples (A–F) in negative and positive scan modes. Dashed lines represent the expected (nominal) values for albiflorin and paeoniflorin and error bars indicate standard deviations of replicate measurements.

## Data Availability

The data that support the findings of this study are available from the corresponding author upon reasonable request.

## References

[bmc70353-bib-0001] Bao, B. , Y. Zhao , H. Gong , S. Shi , H. Wang , and S. Wang . 2022. “Quantification of Paeoniflorin by Fully Validated LC‐MS/MS Method: Its Application to Pharmacokinetic Interaction Between Paeoniflorin and Verapamil.” Molecules (Basel, Switzerland) 27, no. 23: 8337. 10.3390/molecules27238337. 36500431 PMC9737983

[bmc70353-bib-0002] Cech, N. B. , and C. G. Enke . 2001. “Practical Implications of Some Recent Studies in Electrospray Ionization Fundamentals.” Mass Spectrometry Reviews 20, no. 6: 362–387. 10.1002/mas.10008. 11997944

[bmc70353-bib-0003] Dolan, J. W. 2006. “A Guide to HPLC and LC‐MS Buffer Selection.” 6–7. https://www.hplc.eu/Downloads/ACE_Guide_BufferSelection.pdf.

[bmc70353-bib-0004] European Medicines Agency . 2022. “ICH Guideline M10 on Bioanalytical Method Validation and Study Sample Analysis.” 7–17. https://www.ema.europa.eu/en/ich‐m10‐bioanalytical‐method‐validation‐scientific‐guideline.

[bmc70353-bib-0005] Fang, P. , Y. Wang , F. Sun , H. Lin , and X. Zhang . 2023. “Effects of Albiflorin on Oxidative Stress and Inflammatory Responses in Rats With Acute Spinal Cord Injury.” Immunity, Inflammation and Disease 11, no. 9: e1015. 10.1002/iid3.1015. 37773716 PMC10510471

[bmc70353-bib-0006] Gao, L.‐N. , Y. Zhang , Y.‐L. Cui , and O. M. Akinyi . 2015. “Comparison of Paeoniflorin and Albiflorin on Human CYP3A4 and CYP2D6.” Evidence‐Based Complementary and Alternative Medicine: ECAM 2015: 470219. 10.1155/2015/470219. 26089940 PMC4452296

[bmc70353-bib-0007] He, D.‐Y. , and S.‐M. Dai . 2011. “Anti‐Inflammatory and Immunomodulatory Effects of *Paeonia lactiflora* Pall., a Traditional Chinese Herbal Medicine.” Frontiers in Pharmacology 2: 10. 10.3389/fphar.2011.00010. 21687505 PMC3108611

[bmc70353-bib-0008] Ho, S.‐L. , C.‐Y. Poon , C. Lin , et al. 2015. “Inhibition of β‐Amyloid Aggregation by Albiflorin, Aloeemodin and Neohesperidin and Their Neuroprotective Effect on Primary Hippocampal Cells Against β‐Amyloid Induced Toxicity.” Current Alzheimer Research 12, no. 5: 424–433. 10.2174/1567205012666150504144919. 25938872

[bmc70353-bib-0009] Jeon, M.‐H. , H.‐J. Kwon , J.‐S. Jeong , Y.‐M. Lee , and S.‐P. Hong . 2009. “Detection of Albiflorin and Paeoniflorin in Paeoniae Radix by Reversed‐Phase High‐Performance Liquid Chromatography With Pulsed Amperometric Detection.” Journal of Chromatography. A 1216, no. 21: 4568–4573. 10.1016/j.chroma.2009.03.058. 19345952

[bmc70353-bib-0010] Liu, Y. , L. Feng , and L. Yao . 2024. “Albiflorin Alleviates Sepsis‐Induced Acute Liver Injury Through mTOR/p70S6K Pathway.” Current Molecular Medicine 24, no. 3: 344–354. 10.2174/1566524023666230309124004. 36892118

[bmc70353-bib-0011] Lupo, S. , and T. Kahler . 2017. “New Advice on an Old Topic: Buffers in Reversed‐Phase HPLC.” LCGC North America 35: 424–433. https://www.chromatographyonline.com/view/new‐advice‐old‐topic‐buffers‐reversed‐phase‐hplc.

[bmc70353-bib-0012] Ma, X. , M. Song , Y. Yan , et al. 2021. “Albiflorin Alleviates Cognitive Dysfunction in STZ‐Induced Rats.” Aging 13, no. 14: 18287–18297. 10.18632/aging.203274. 34319254 PMC8351685

[bmc70353-bib-0013] Park, C. , H.‐J. Cha , D.‐G. Kim , et al. 2025. “Albiflorin, a Monoterpene Glycoside, Protects Myoblasts Against Hydrogen Peroxide‐Induced Apoptosis by Activating the Nrf2/HO‐1 Axis.” Biomolecules & Therapeutics 33, no. 4: 716–727. 10.4062/biomolther.2025.020. 40492376 PMC12215032

[bmc70353-bib-0014] Sheng, Y. , L. Li , J. Zhang , and D. Guo . 2004. “Simultaneous Determination of Albiflorin and Paeoniflorin in Rat Urine by Solid‐Phase Extraction and High‐Performance Liquid Chromatography Following Oral Administration of Si‐Wu Decoction.” Biomedical Chromatography: BMC 18, no. 10: 785–790. 10.1002/bmc.389. 15386578

[bmc70353-bib-0015] Shi, Y.‐H. , S. Zhu , Y.‐W. Ge , et al. 2016. “Monoterpene Derivatives With Anti‐Allergic Activity From Red Peony Root, the Root of *Paeonia lactiflora* .” Fitoterapia 108: 55–61. 10.1016/j.fitote.2015.11.011. 26598138

[bmc70353-bib-0016] Shimadzu Deutschland GmbH . Mobile Phases Compatible for LCMS. https://www.shimadzu.de/service‐support/technical‐support/analysis‐basics/basics_of_lcms/compatible_for_lcms.html.

[bmc70353-bib-0017] Song, J. , B.‐F. Qin , Q.‐Y. Feng , et al. 2024. “Albiflorin Ameliorates Thioacetamide‐Induced Hepatic Fibrosis: The Involvement of NURR1‐Mediated Inflammatory Signaling Cascades in Hepatic Stellate Cells Activation.” Ecotoxicology and Environmental Safety 276: 116334. 10.1016/j.ecoenv.2024.116334. 38626607

[bmc70353-bib-0018] Tan, Y.‐Q. , H.‐W. Chen , J. Li , and Q.‐J. Wu . 2020. “Efficacy, Chemical Constituents, and Pharmacological Actions of Radix Paeoniae Rubra and Radix Paeoniae Alba.” Frontiers in Pharmacology 11: 1054. 10.3389/fphar.2020.01054. 32754038 PMC7365904

[bmc70353-bib-0019] Tong, L. , M. Wan , D. Zhou , J. Gao , Y. Zhu , and K. Bi . 2010. “Lc‐MS/MS Determination and Pharmacokinetic Study of Albiflorin and Paeoniflorin in Rat Plasma After Oral Administration of Radix Paeoniae Alba Extract and Tang‐Min‐Ling‐Wan.” Biomedical Chromatography: BMC 24, no. 12: 1324–1331. 10.1002/bmc.1443. 21077251

[bmc70353-bib-0020] Wang, Q.‐S. , T. Gao , Y.‐L. Cui , L.‐N. Gao , and H.‐L. Jiang . 2014. “Comparative Studies of Paeoniflorin and Albiflorin From *Paeonia lactiflora* on Anti‐Inflammatory Activities.” Pharmaceutical Biology 52, no. 9: 1189–1195. 10.3109/13880209.2014.880490. 24646307

[bmc70353-bib-0021] Xu, Y.‐J. , Y. Mei , X.‐Q. Shi , et al. 2019. “Albiflorin Ameliorates Memory Deficits in APP/PS1 Transgenic Mice via Ameliorating Mitochondrial Dysfunction.” Brain Research 1719: 113–123. 10.1016/j.brainres.2019.05.037. 31150651

[bmc70353-bib-0022] Zhang, X.‐X. , J.‐Q. Zuo , Y.‐T. Wang , H.‐Y. Duan , J.‐H. Yuan , and Y.‐H. Hu . 2022. “Paeoniflorin in Paeoniaceae: Distribution, Influencing Factors, and Biosynthesis.” Frontiers in Plant Science 13: 980854. 10.3389/fpls.2022.980854. 36119574 PMC9478390

[bmc70353-bib-0023] Zhu, Y. , L. Wang , Z. Yang , et al. 2016. “Hematopoietic Effects of Paeoniflorin and Albiflorin on Radiotherapy‐Induced Myelosuppression Mice.” Evidence‐Based Complementary and Alternative Medicine: ECAM 2016: 5789381. 10.1155/2016/5789381. 27313650 PMC4899601

