# Supplementary material for: A Buffered LC‐MS Method for Resolving and Quantifying Albiflorin and Paeoniflorin
Source: Biomed Chromatogr. 2026 Jan 18;40(3):e70353. doi: 10.1002/bmc.70353 (PMC12813524; doi:10.1002/bmc.70353)
Supplement: Supplementary file 1 — Data S1: Supporting information. [file BMC-40-e70353-s002.docx]

A Buffered LC-MS Method for Resolving and Quantifying Albiflorin and Paeoniflorin
Supporting Information

Alina Gazizova^a^, Ela Hümay Altincubuk, Christina Oppermann^a,*^

a Industrial and Analytical Chemistry, Department of Chemistry, University of Rostock, Albert-Einstein-Straße 3a, 18059 Rostock

* Corresponding author at: Industrial and Analytical Chemistry, Department of Chemistry, University of Rostock, Albert-Einstein-Straße 3a, 18059 Rostock, Germany
Tel.: + 49 (0)381/498-64506453; E‑mail address: christina.oppermann@uni-rostock.de

Table of Contents

[1. Mass Spectra 2](#_Toc210137679)

[1.1. Comparison of mass spectra of albiflorin and paeoniflorin standards measured with non-buffered and buffered eluent systems. 2](#_Toc210137680)

[2. NMR spectra 3](#_Toc210137681)

[2.1. Deuterated dimethyl sulfoxide (DMSO-d6) as solvent 3](#_Toc210137682)

[2.1.1. ^13^C 3](#_Toc210137683)

[2.1.2. DEPT 4](#_Toc210137684)

[2.1.3. COSY 5](#_Toc210137685)

[2.1.4. ^1^H-^13^C-HSQC 6](#_Toc210137686)

[2.1.5. ^1^H-^13^C-HMBC 7](#_Toc210137687)

[2.1.6. NOESY 8](#_Toc210137688)

[2.1.7. TOCSY 9](#_Toc210137689)

[2.1.8. Comparison of ^1^H-NMR after addition of 1 µl and 10 µl formic acid. 10](#_Toc210137690)

[2.2. Deuterated methanol (MeOD-d4) as solvent 11](#_Toc210137691)

[2.2.1. ^1^H-NMR 11](#_Toc210137692)

[2.2.2. ^13^C 12](#_Toc210137693)

[2.2.3. DEPT 13](#_Toc210137694)

[2.2.4. COSY 14](#_Toc210137695)

[2.2.5. ^1^H-^13^C-HSQC 15](#_Toc210137696)

[2.2.6. ^1^H-^13^C-HMBC 16](#_Toc210137697)

[2.2.7. Comparison of ^1^H-NMR after addition of 1 µl and 10 µl formic acid. 17](#_Toc210137698)

[3. Calibration and Method Validation 18](#_Toc210137699)

[3.1. Calibration curves in positive and negative scan mode 18](#_Toc210137700)

[3.2. Method validation samples 19](#_Toc210137701)

# Mass Spectra

## Comparison of mass spectra of albiflorin and paeoniflorin standards measured with non-buffered and buffered eluent systems.


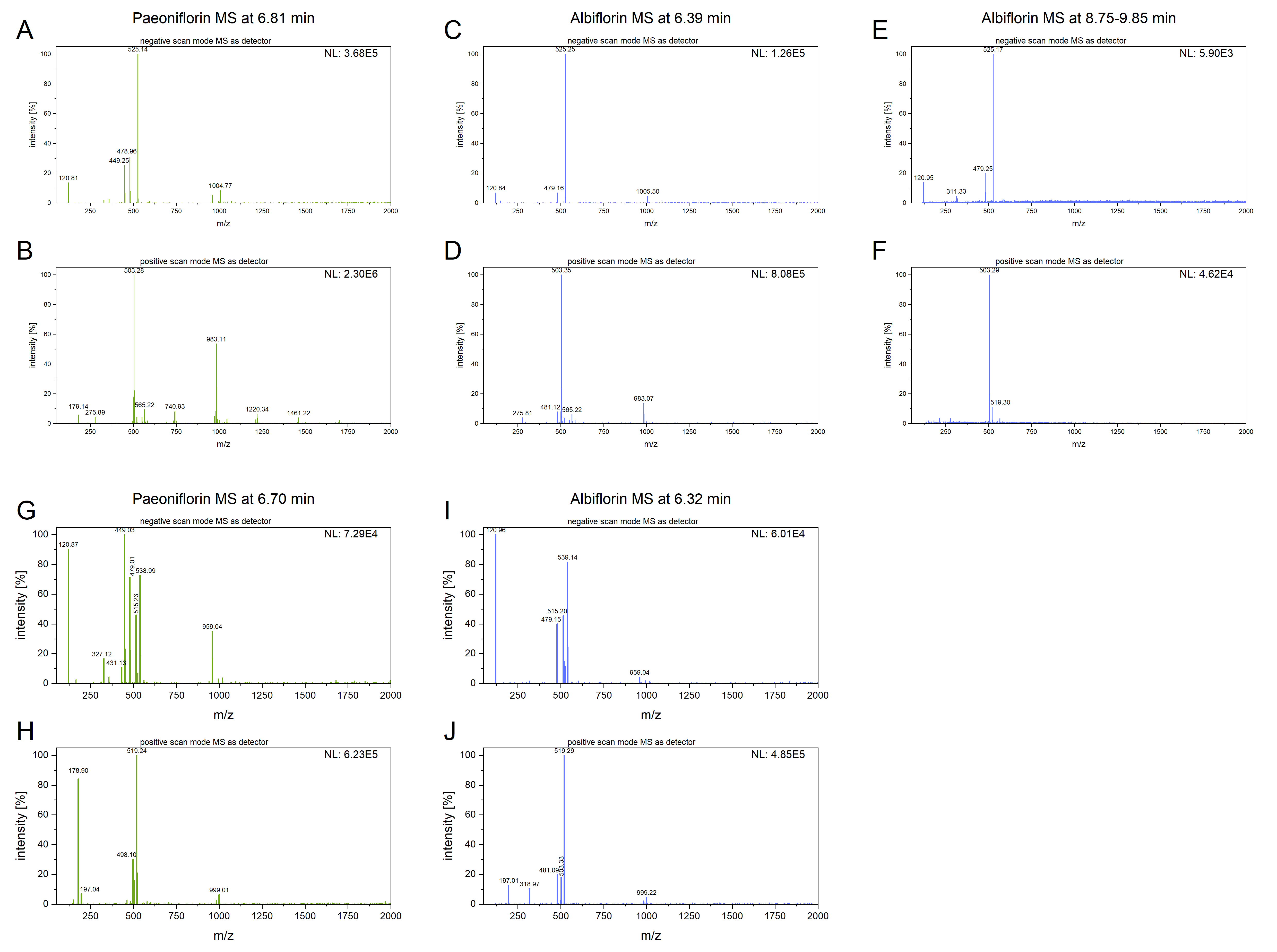


Supporting Figure 1 Mass spectra of paeoniflorin and albiflorin standards in negative and positive scan mode. Spectra A-F were obtained using the non-buffered eluent system and spectra G-J were measured using the buffered eluent system. A, B, G and H show paeoniflorin, while C, D, I and J show albiflorin. E and F display the mass spectrum of the second peak found in the chromatogram of the albiflorin standard measured with method 1.

# NMR spectra

## Deuterated dimethyl sulfoxide (DMSO-d6) as solvent

- - 1. ^13^C


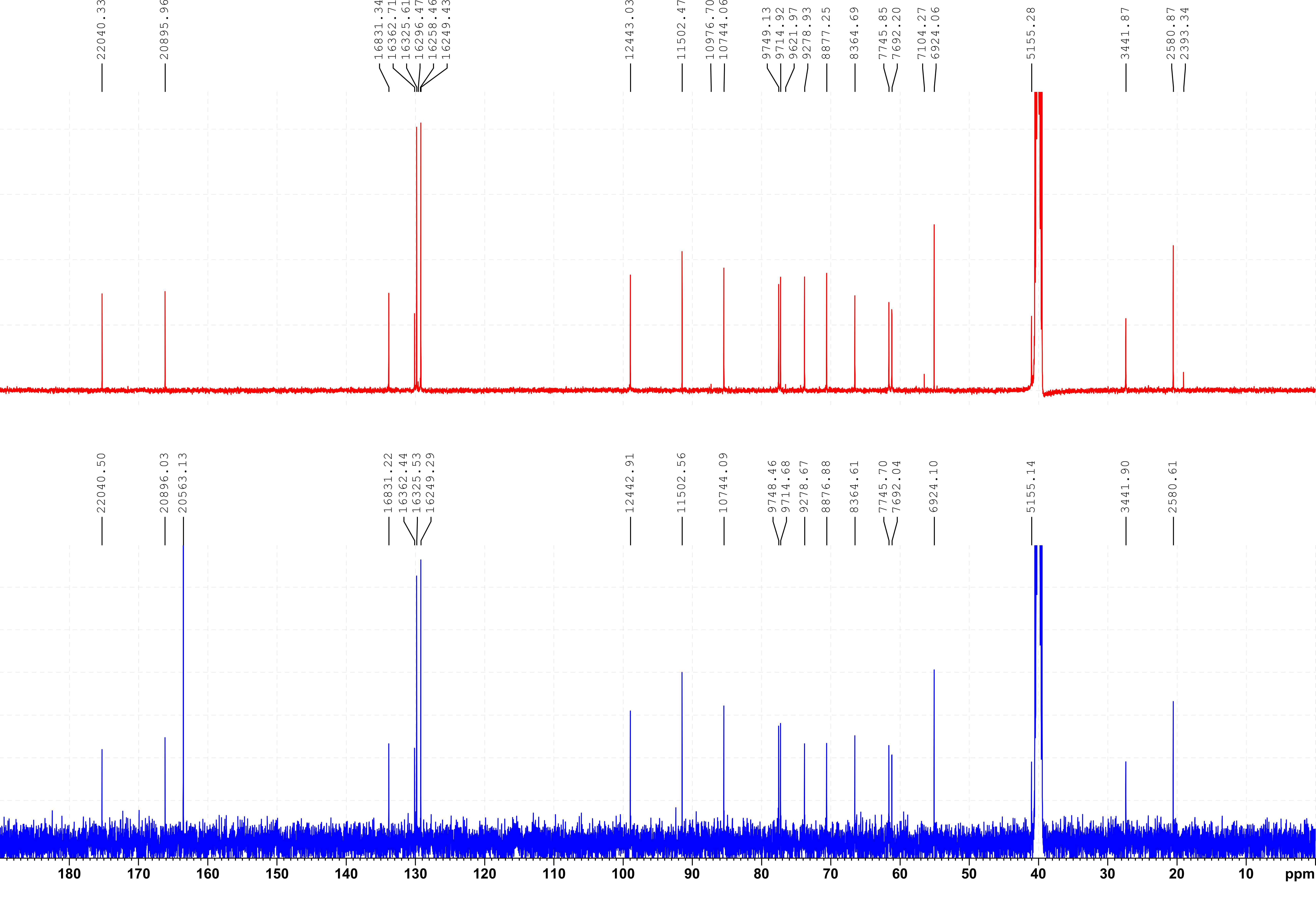


Supporting Figure 2 ^13^C-NMR spectrum of albiflorin standard before (top, red) and after (bottom, blue) formic acid addition.

- - 1. DEPT


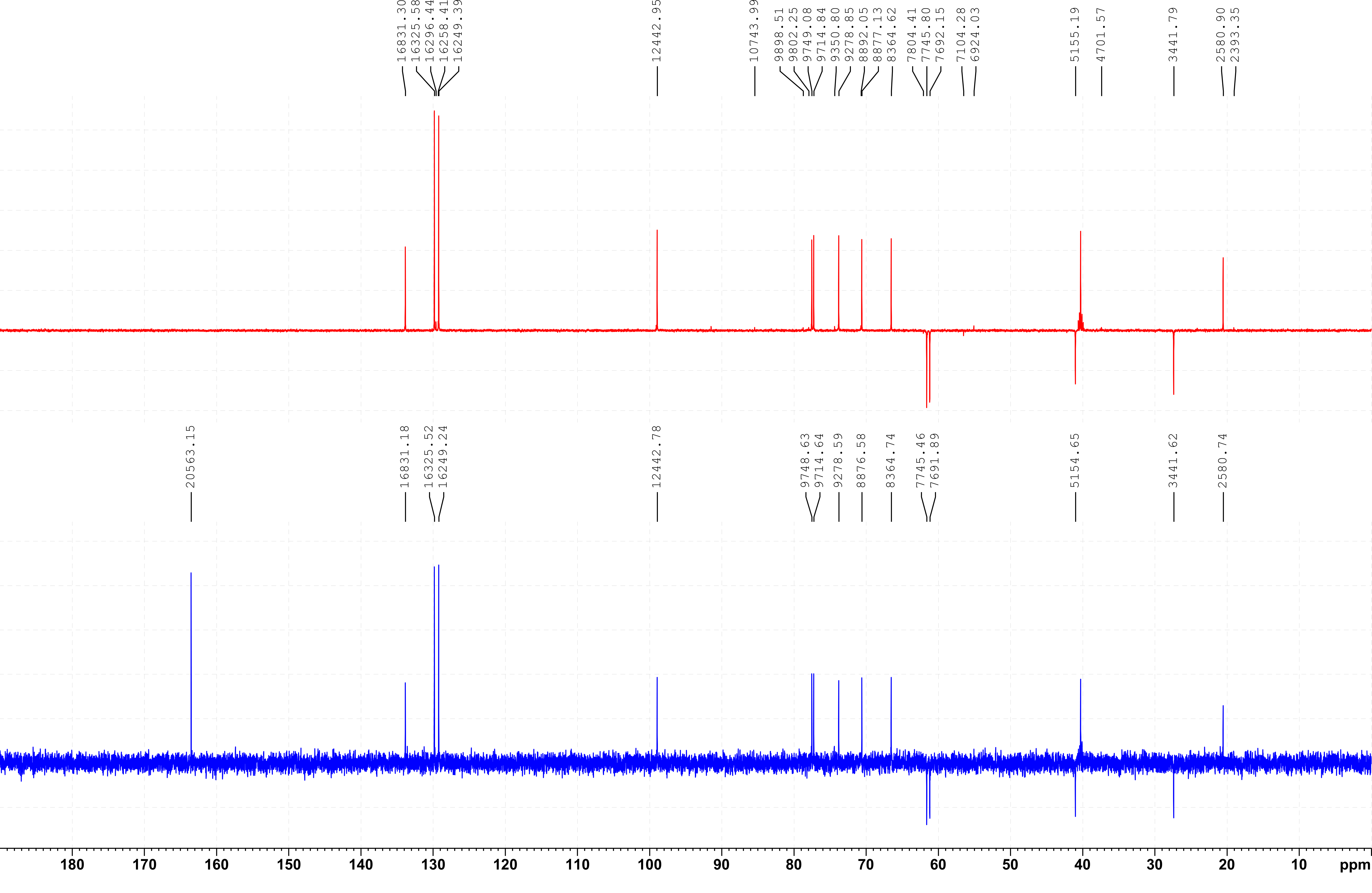


Supporting Figure 3 DEPT-NMR spectrum of albiflorin standard before (top, red) and after (bottom, blue) formic acid addition.

- - 1. COSY


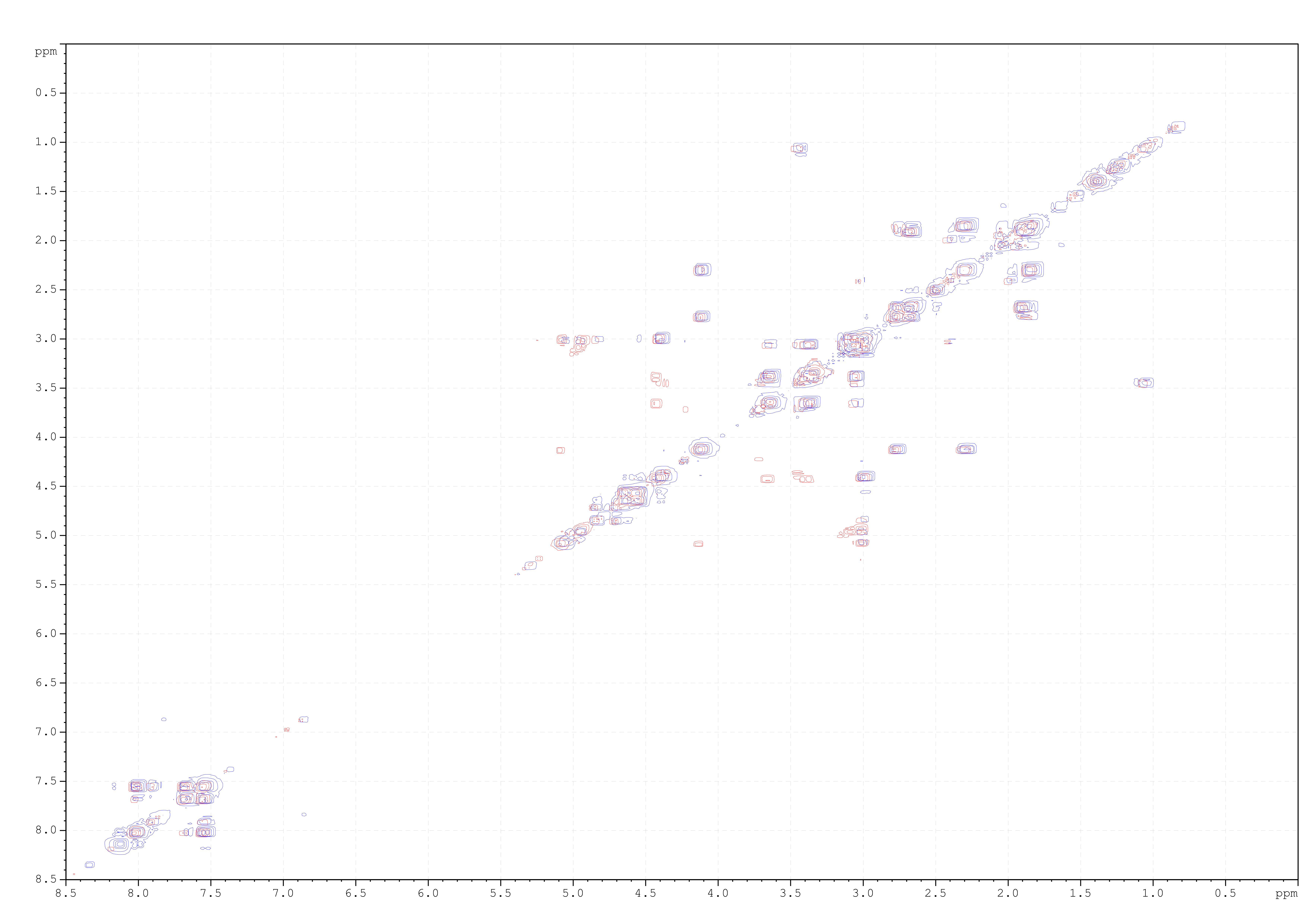


Supporting Figure 4 COSY-NMR spectrum of albiflorin standard before (red) and after (blue) formic acid addition.

- - 1. ^1^H-^13^C-HSQC


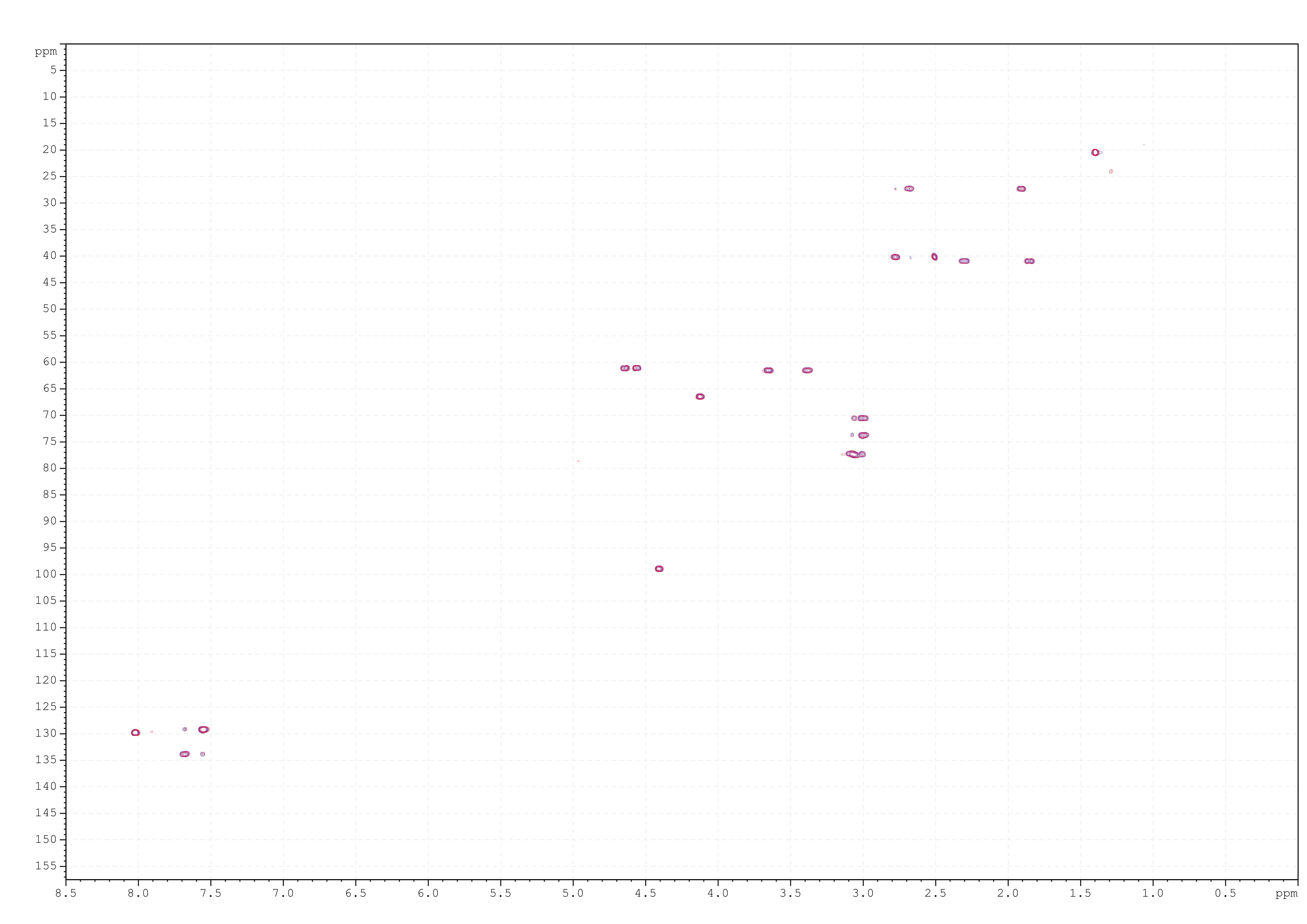


Supporting Figure 5 ^1^H-^13^C-HSQC spectrum of albiflorin standard before (red) and after (blue) formic acid addition.

- - 1. ^1^H-^13^C-HMBC


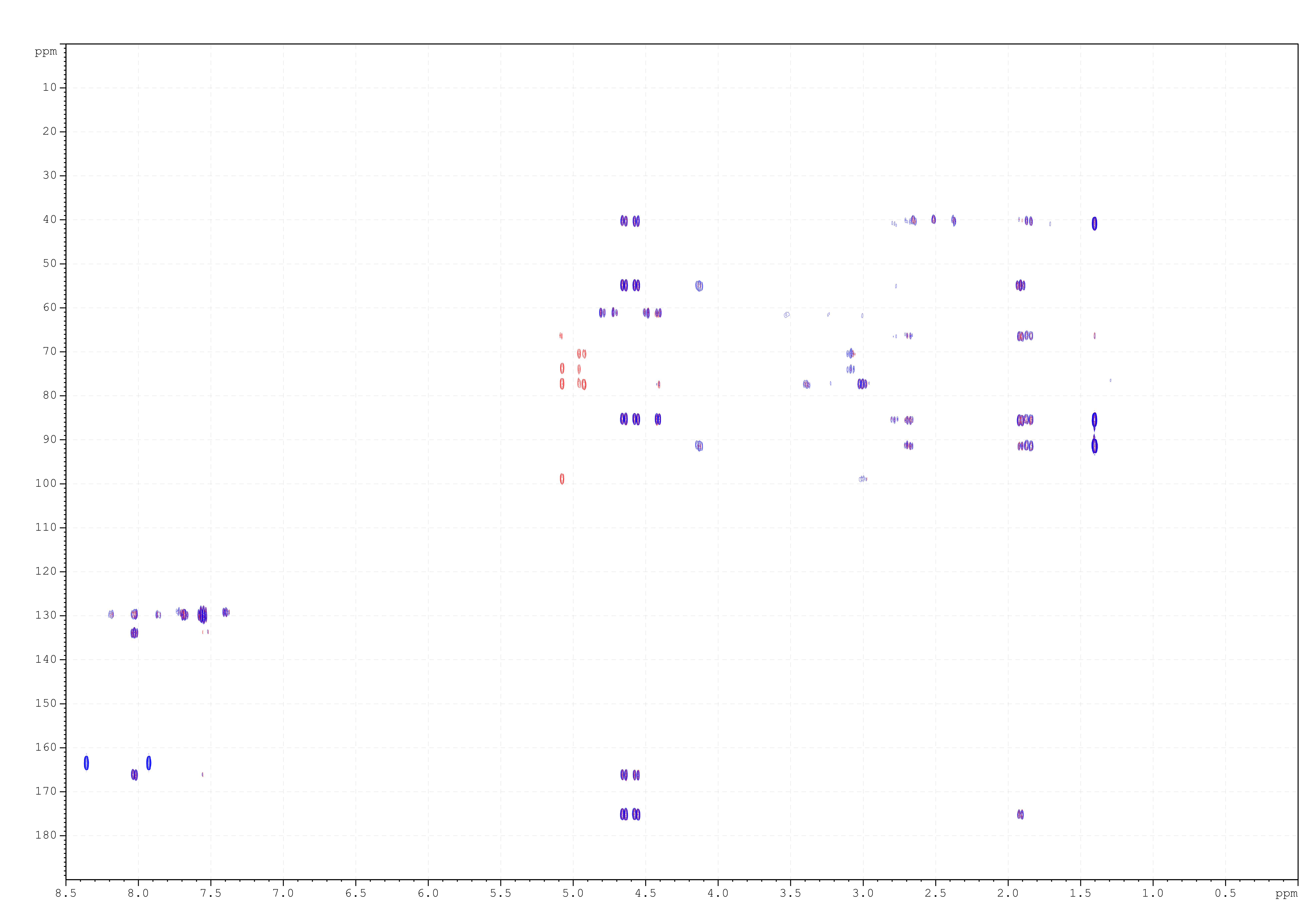


Supporting Figure 6 ^1^H-^13^C-HMBC spectrum of albiflorin standard before (red) and after (blue) formic acid addition.

- - 1. NOESY


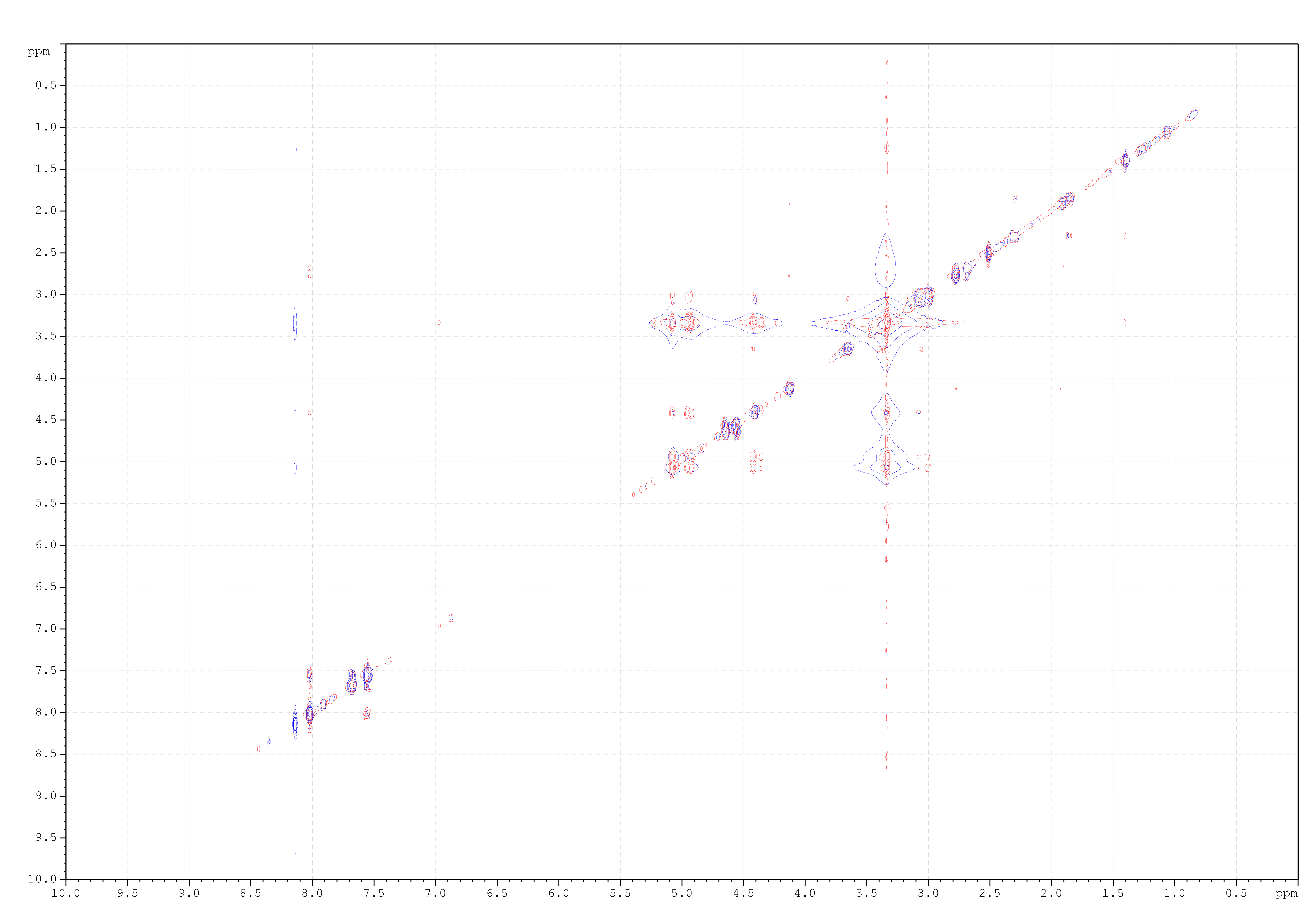


Supporting Figure 7 NOESY spectrum of albiflorin standard before (red) and after (blue) formic acid addition.

- - 1. TOCSY


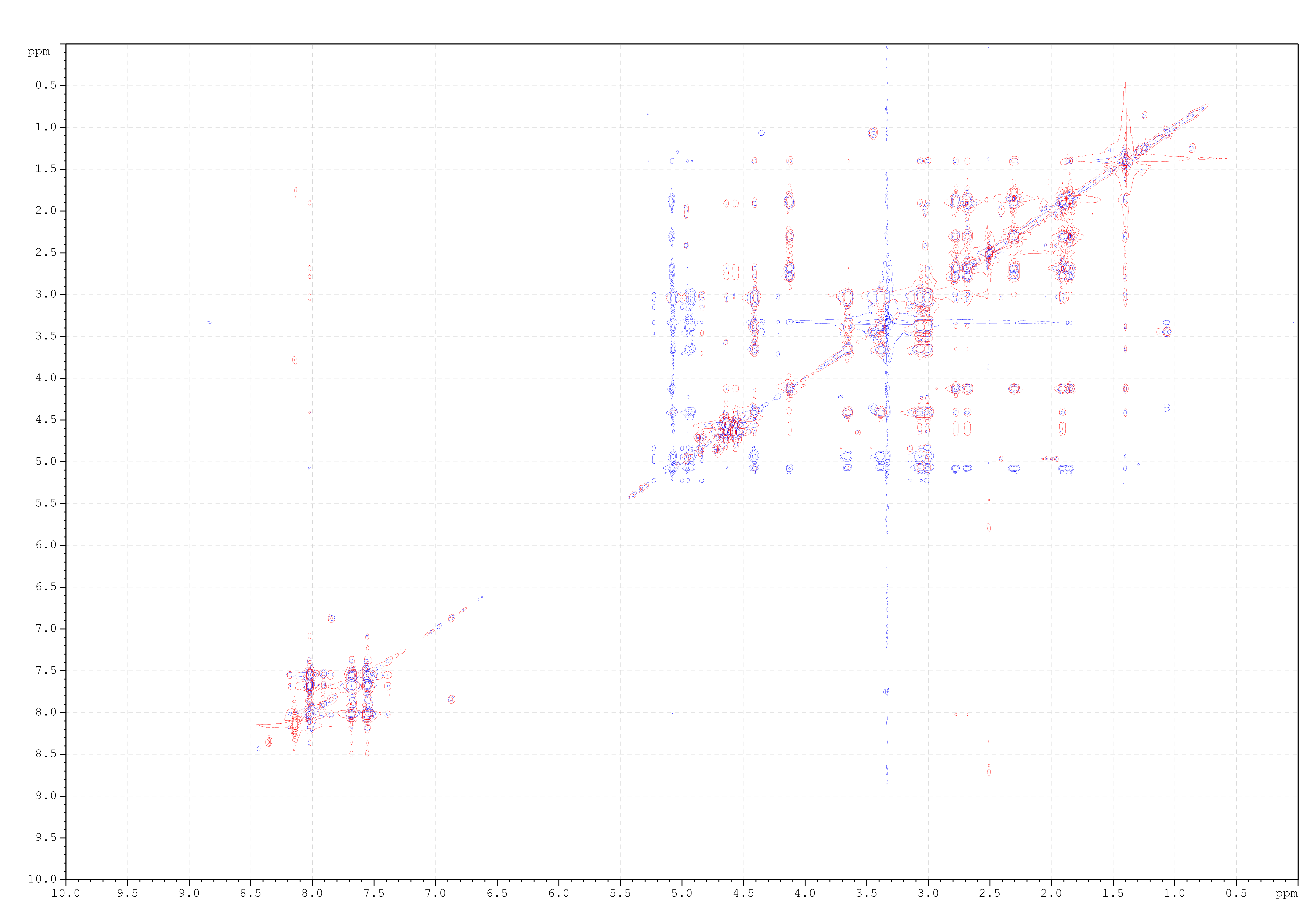


Supporting Figure 8 TOCSY spectrum of albiflorin standard before (red) and after (blue) formic acid addition.

- - 1. Comparison of ^1^H-NMR after addition of 1 µl and 10 µl formic acid.


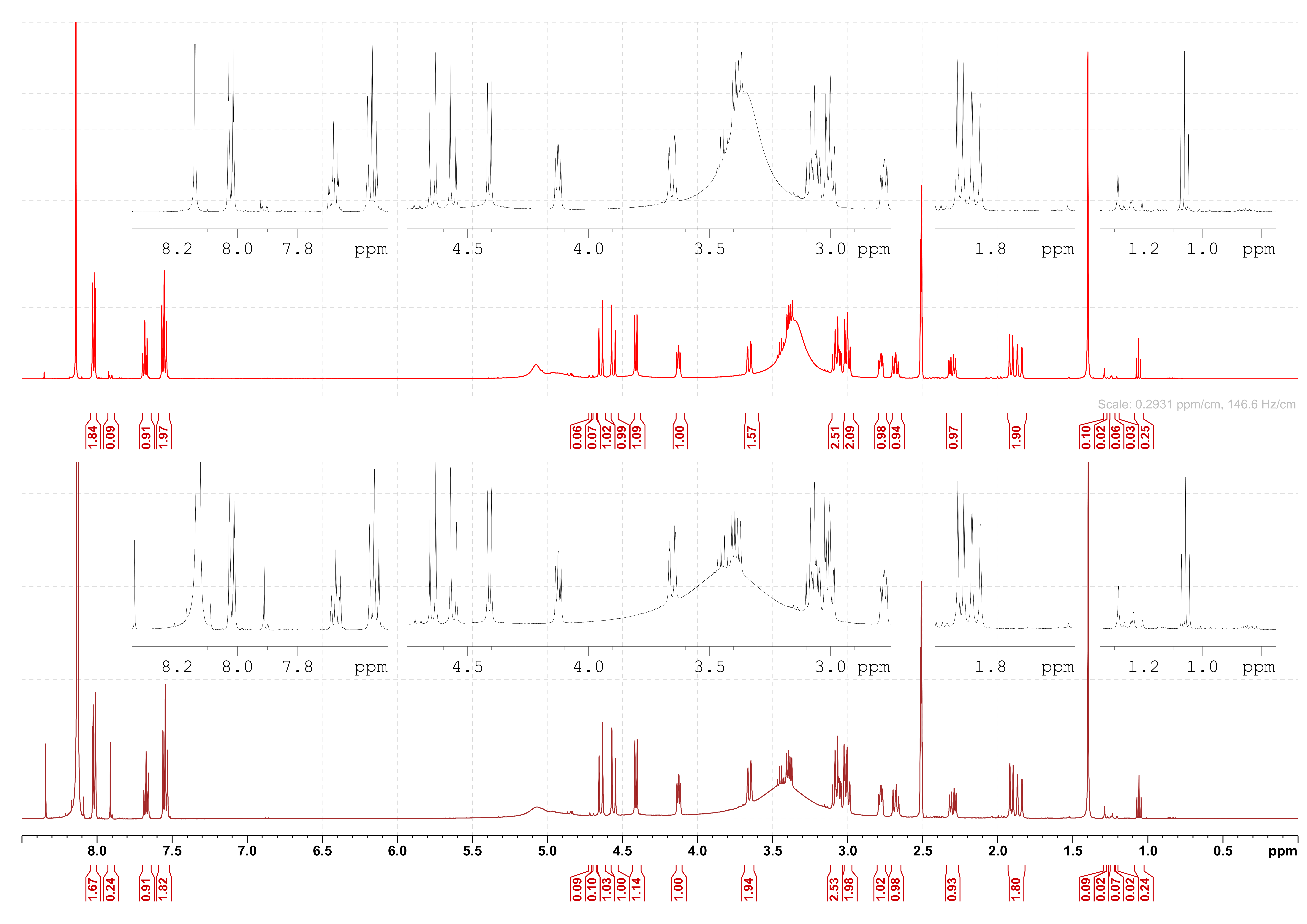


Supporting Figure 9 ^1^H-NMR spectrum of albiflorin after addition of 1 µl (top, red) and 10 µl (bottom, dark red) of formic acid.

## Deuterated methanol (MeOD-d4) as solvent

- - 1. ^1^H-NMR


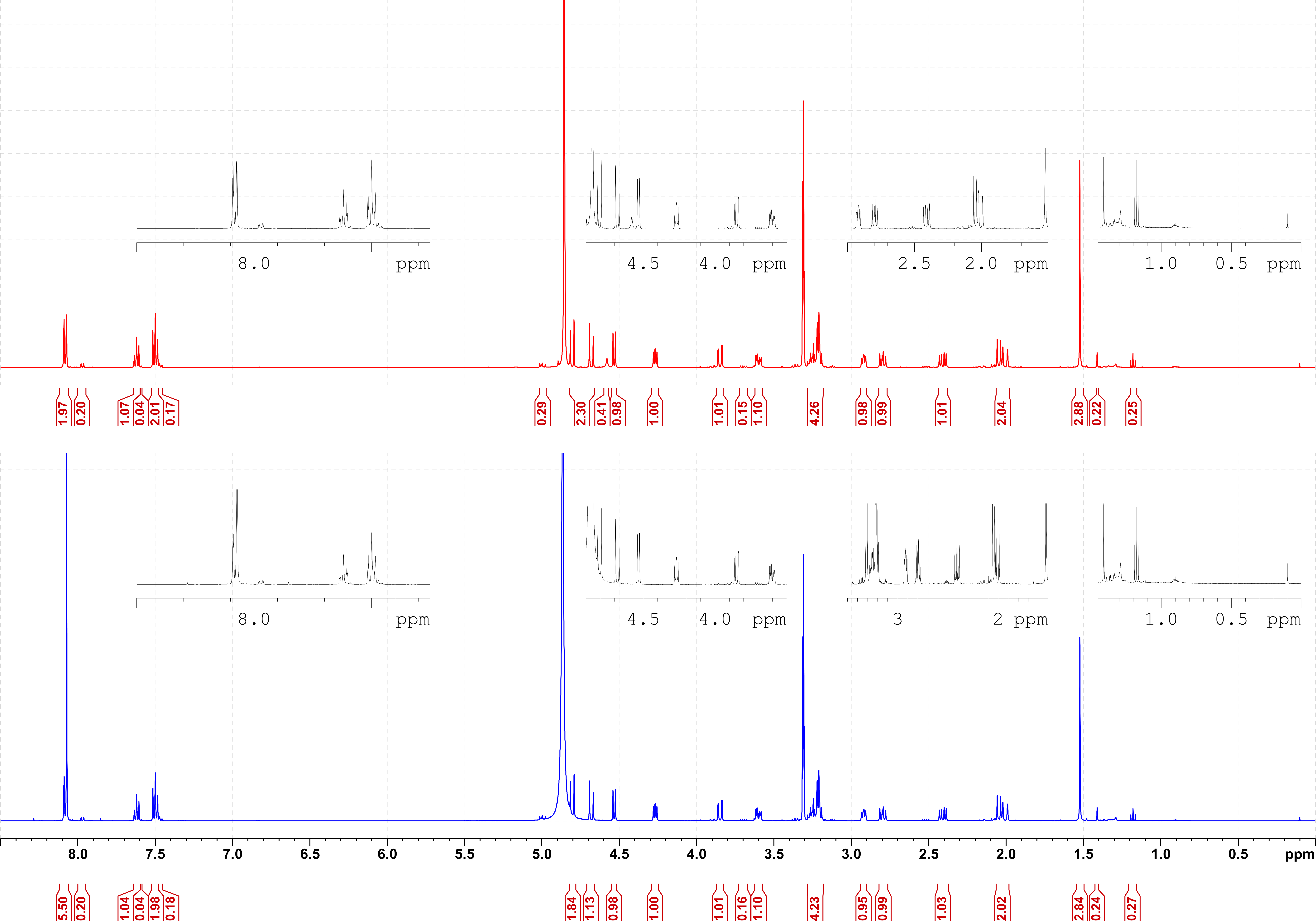


Supporting Figure 10 1H-NMR spectrum of albiflorin before (top, red) and affter (bottom, blue) addition of formic acid.

- - 1. ^13^C


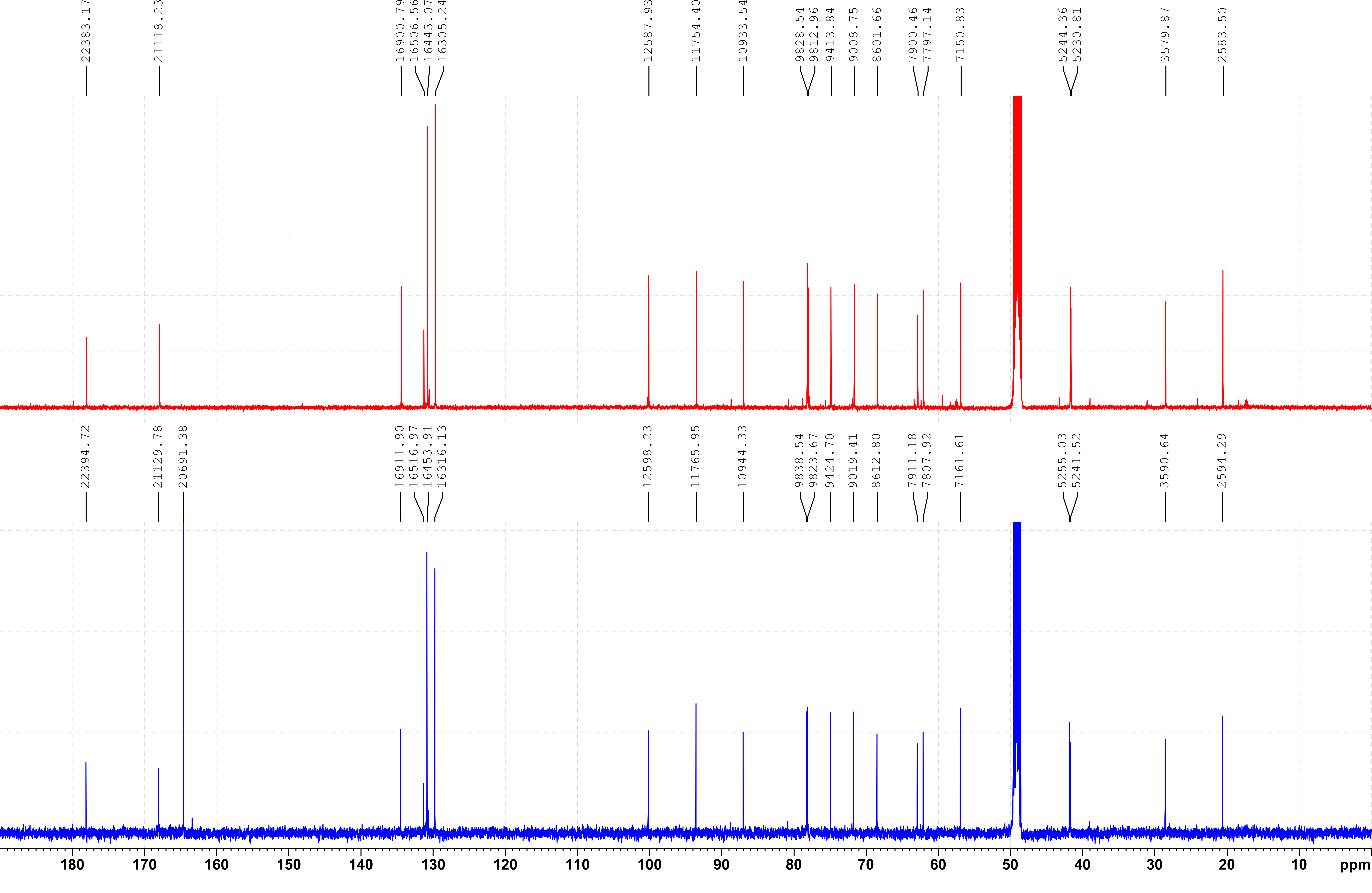


Supporting Figure 11 ^13^C-NMR spectrum of albiflorin standard before (top, red) and after (bottom, blue) formic acid addition.

- - 1. DEPT


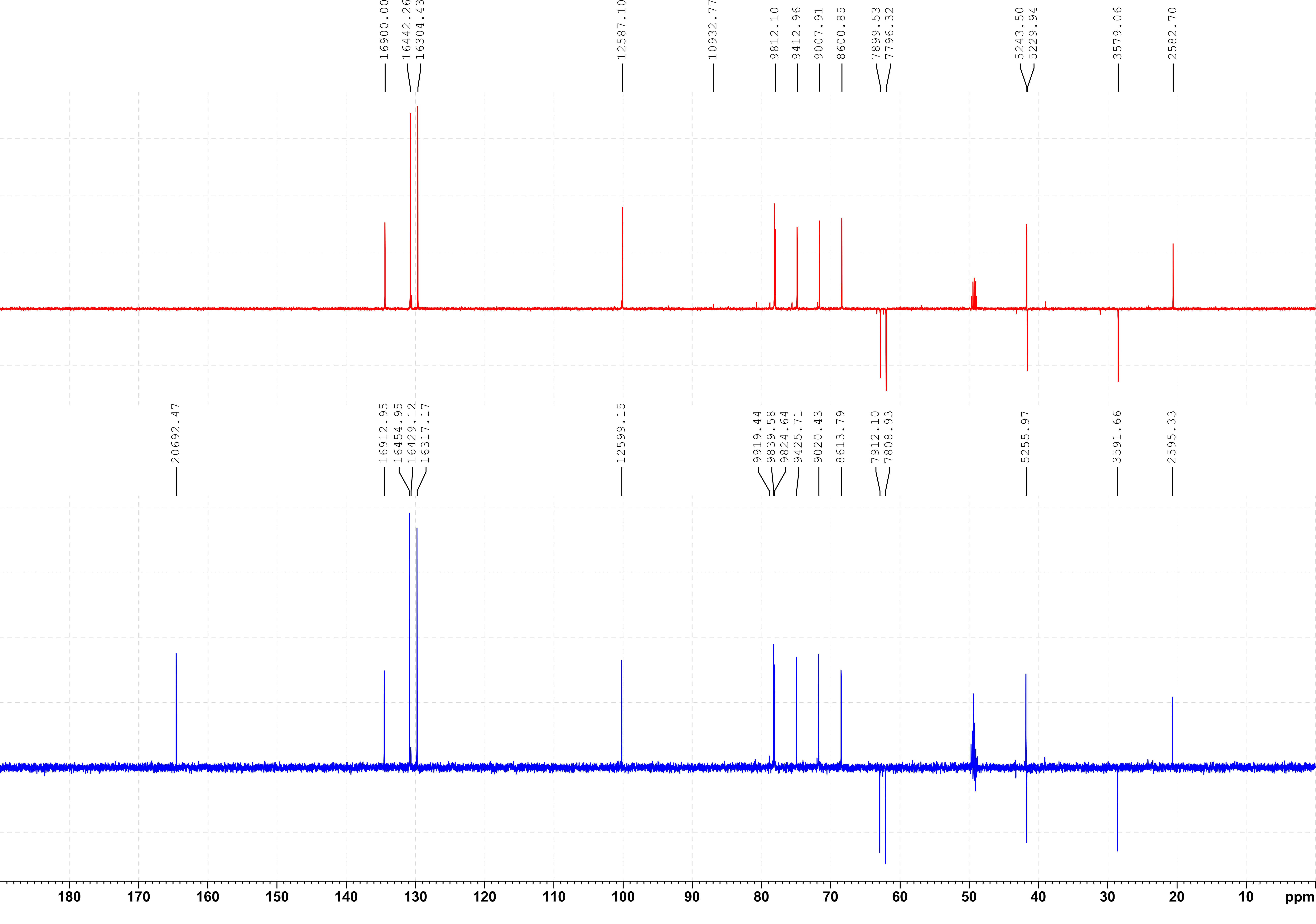


Supporting Figure 12 DEPT-NMR spectrum of albiflorin standard before (top, red) and after (bottom, blue) formic acid addition.

- - 1. COSY


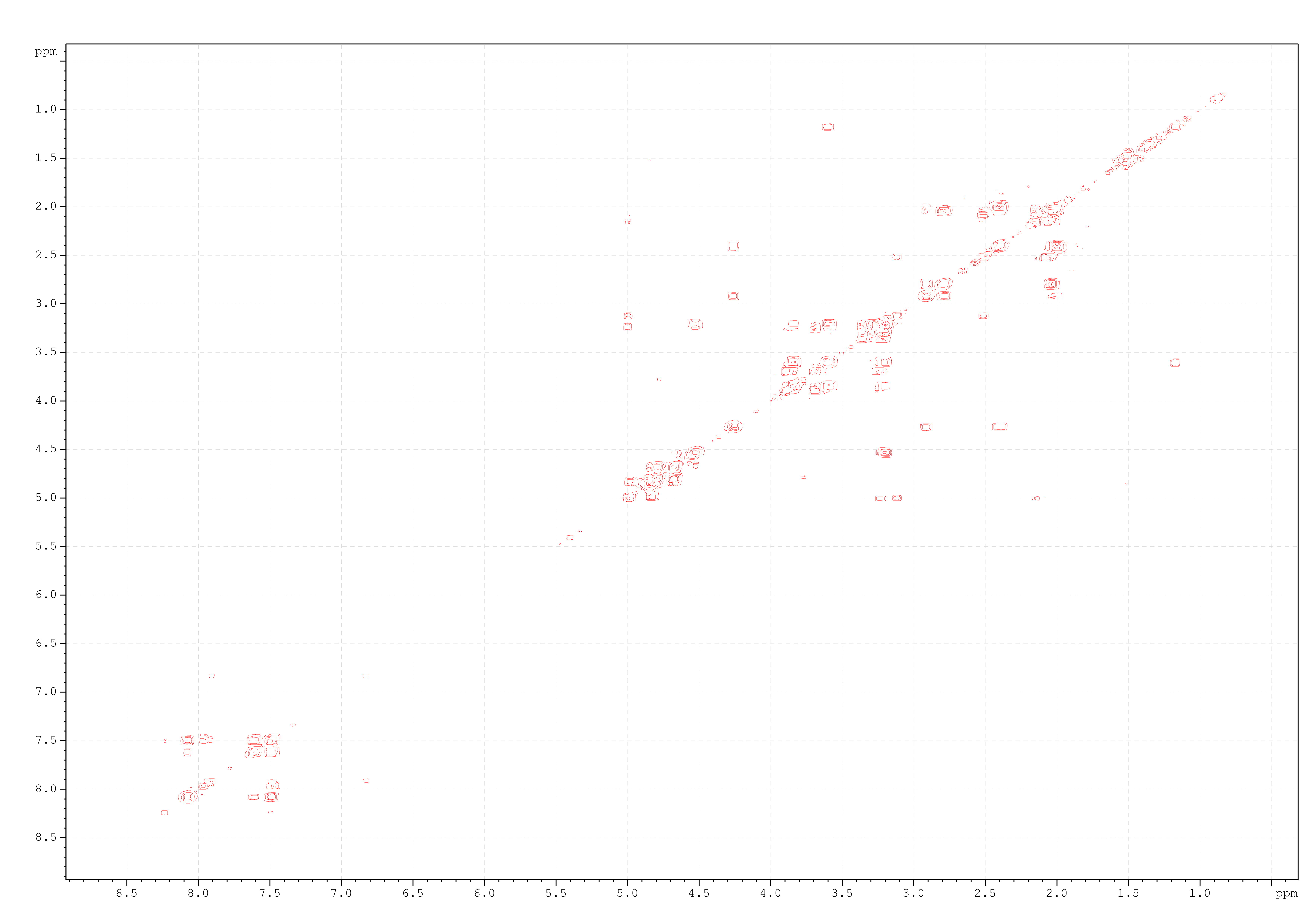


Supporting Figure 13 COSY-NMR spectrum of albiflorin standard after formic acid addition.

- - 1. ^1^H-^13^C-HSQC


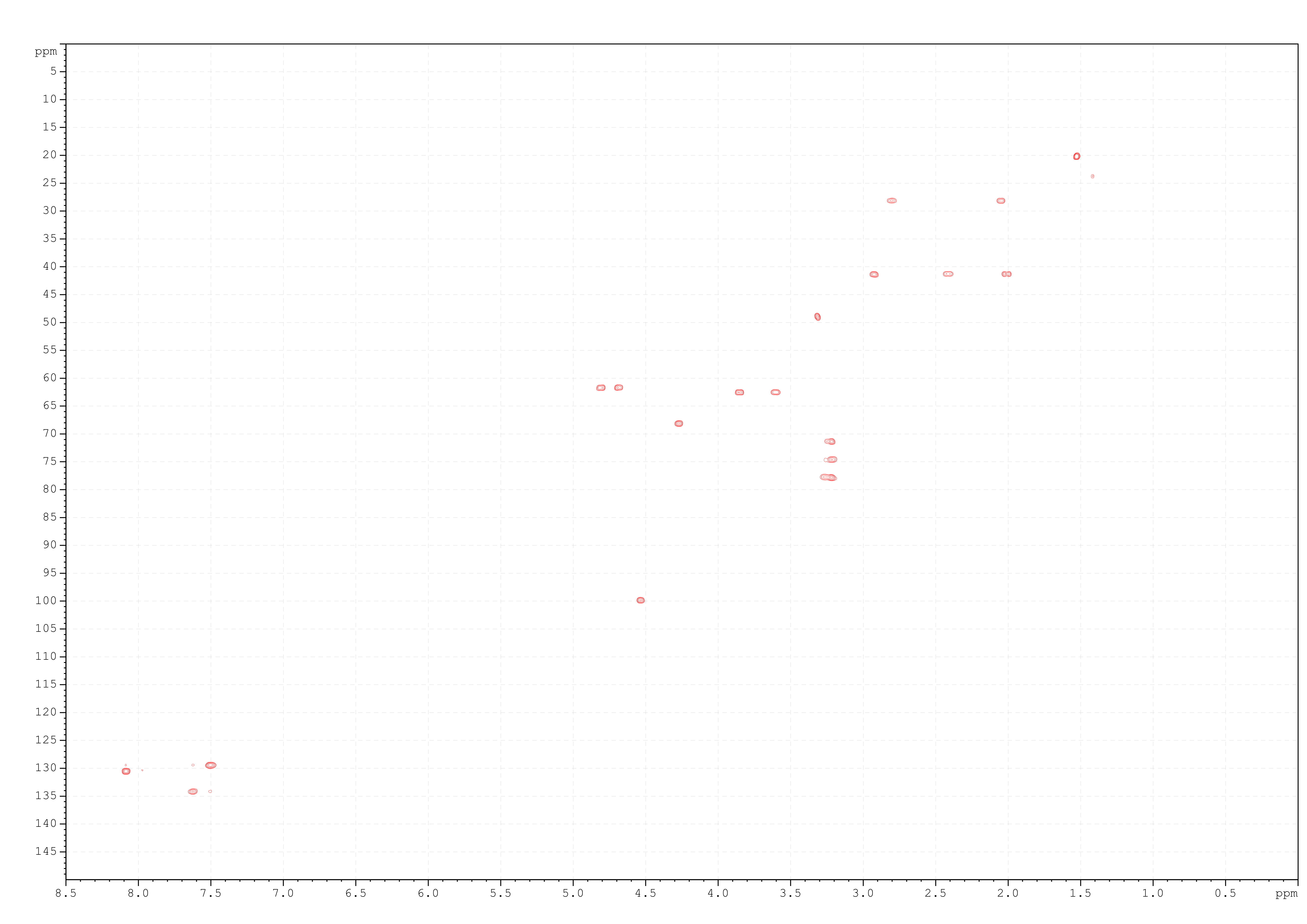


Supporting Figure 14 ^1^H-^13^C-HSQC spectrum of albiflorin standard after formic acid addition.

- - 1. ^1^H-^13^C-HMBC


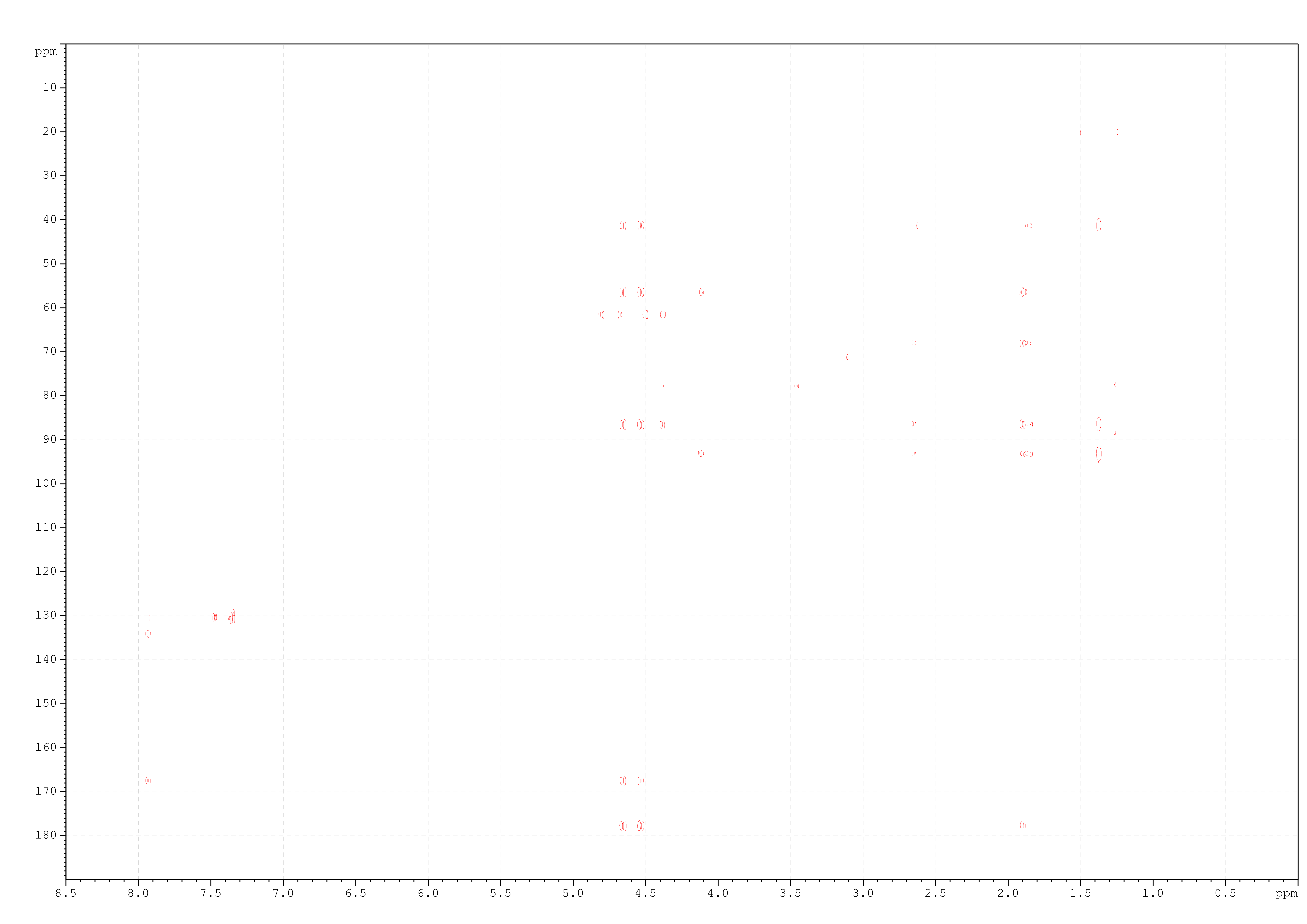


Supporting Figure 15 1H-13C-HMBC spectrum of albiflorin standard after formic acid addition.

- - 1. Comparison of ^1^H-NMR after addition of 1 µl and 10 µl formic acid.


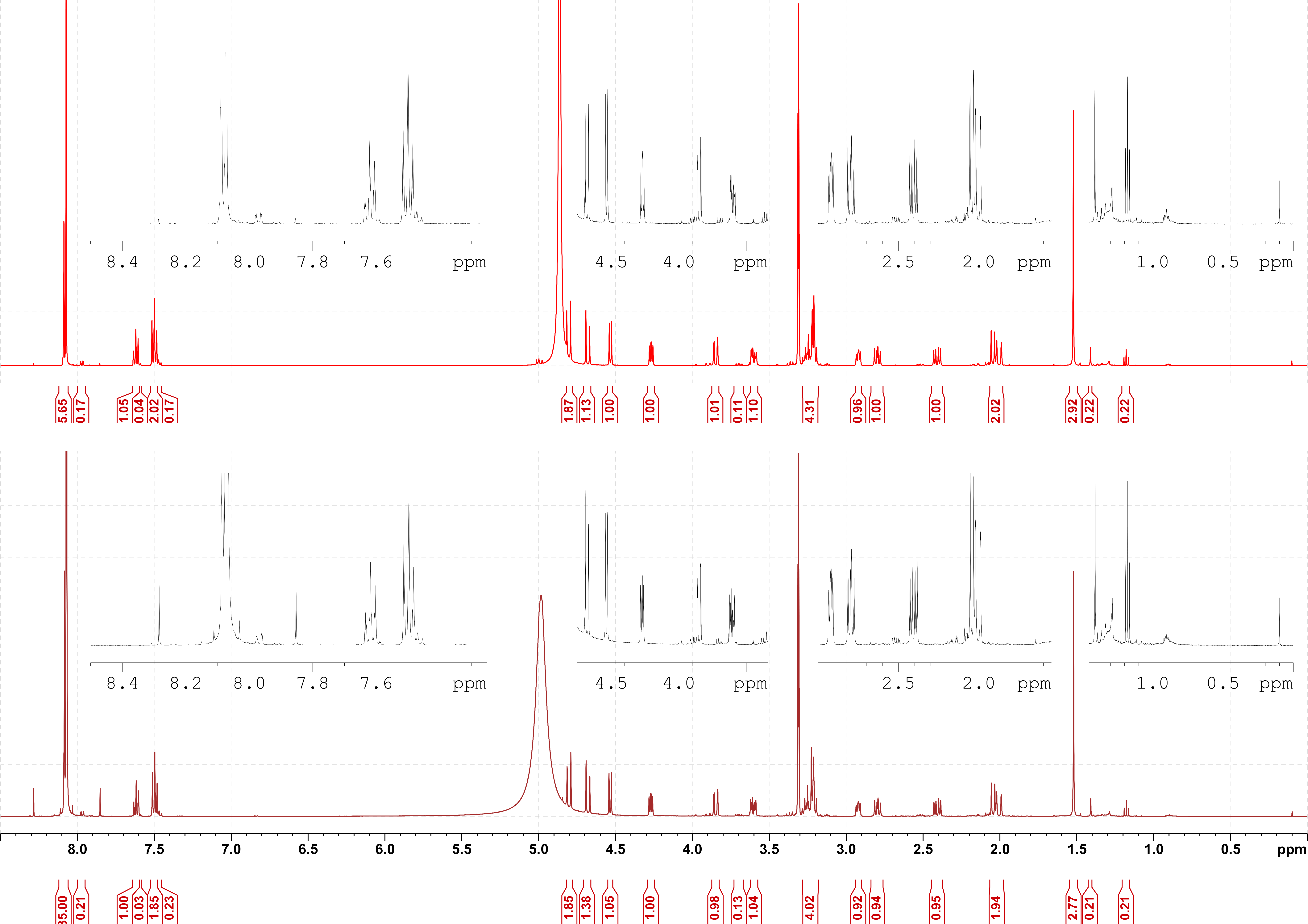


Supporting Figure 16 ^1^H-NMR spectrum of albiflorin after addition of 1 µl (top, red) and 10 µl (bottom, dark red) of formic acid.

# Calibration and Method Validation

## Calibration Curves


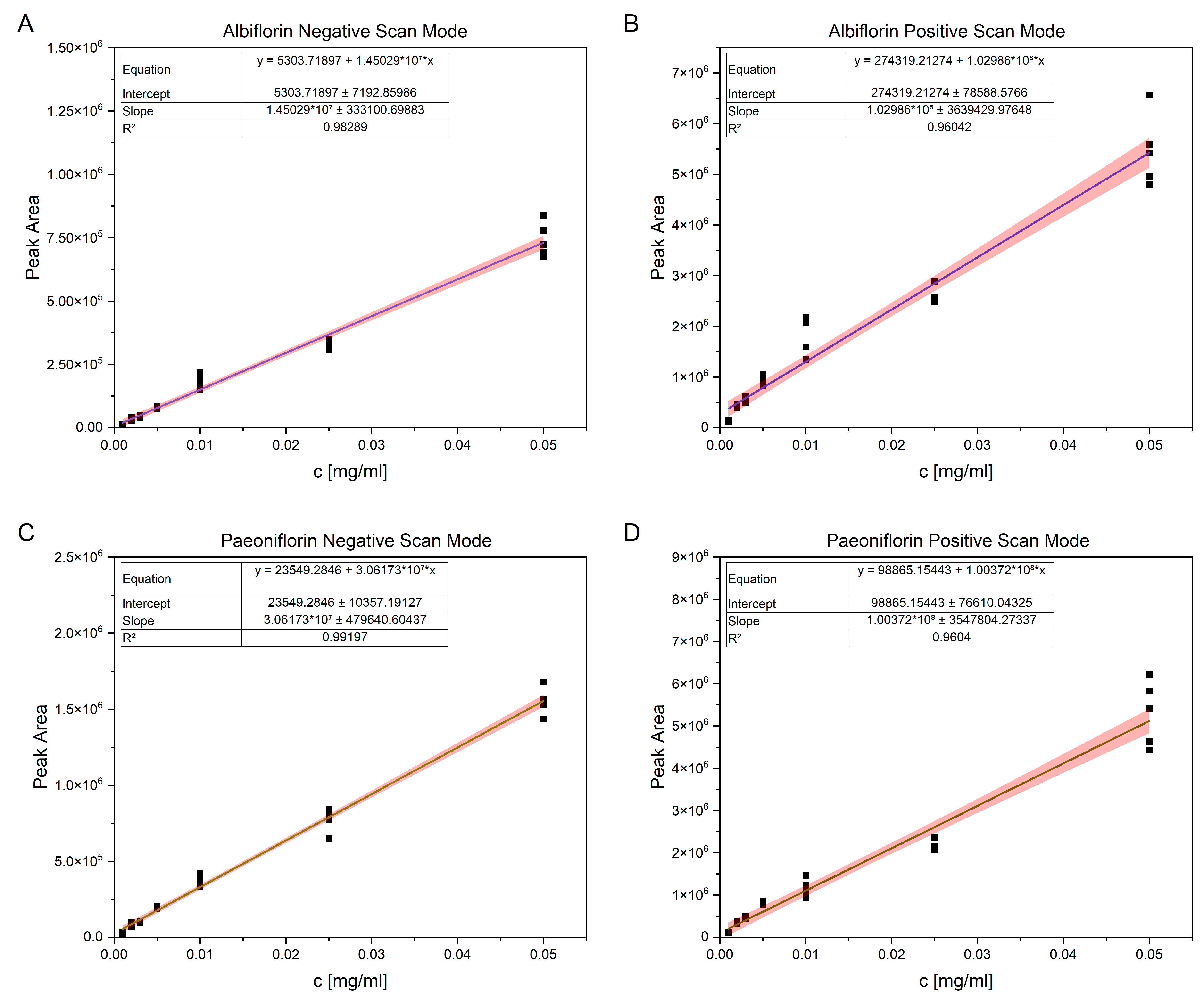


Supporting Figure 17 Calibration curves of albiflorin (A, B) and paeoniflorin (C, D) in negative and positive scan modes. The fitted calibration equations, coefficients of determination (R^2^) and 95% confidence bands are shown.

## Method Validation Samples


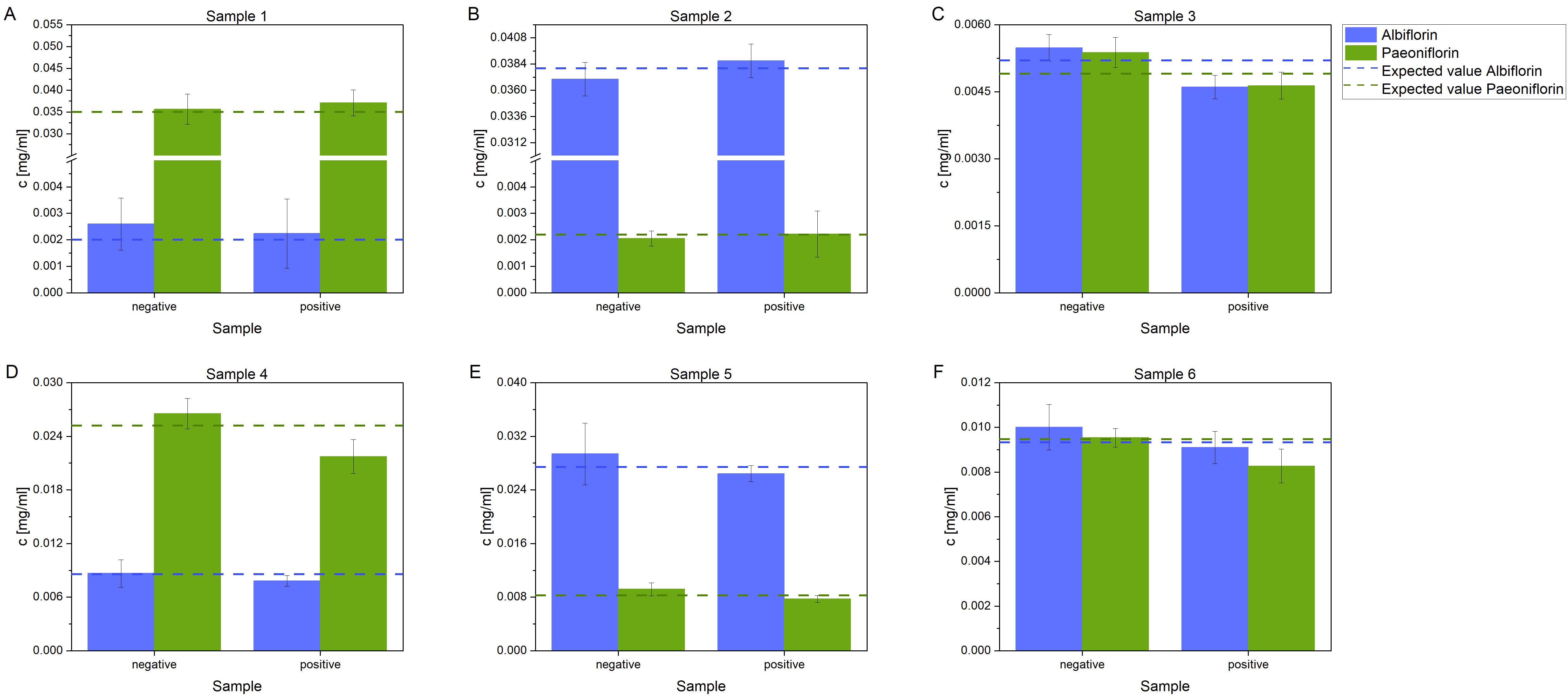


Supporting Figure 18 Measured concentrations of albiflorin (blue) and paeoniflorin (green) in the six validation samples (A-F) in negative and positive scan modes. Dashed lines represent the expected (nominal) values for albiflorin and paeoniflorin and error bars indicate standard deviations of replicate measurements.
