# Supplementary figures and images for: A Buffered LC‐MS Method for Resolving and Quantifying Albiflorin and Paeoniflorin
Source: Biomed Chromatogr. 2026 Jan 18;40(3):e70353. doi: 10.1002/bmc.70353 (PMC12813524; doi:10.1002/bmc.70353)

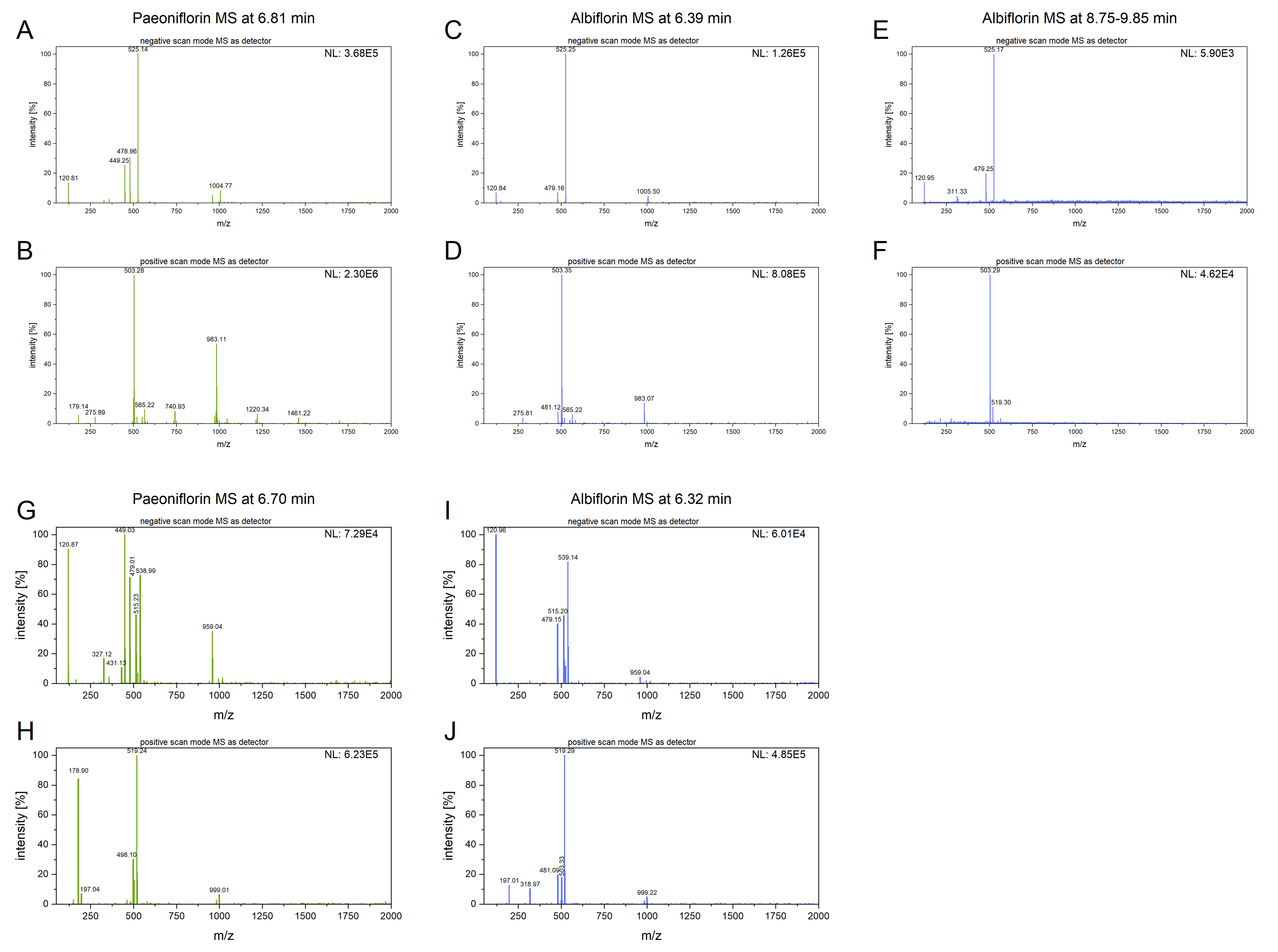

Supplement: Supplementary file 2 — Figure S1: Mass spectra of paeoniflorin and albiflorin standards in negative and positive scan mode. Spectra A–F were obtained using the nonbuffered eluent system and spectra G–J were measured using the buffered eluent system. A, B, G, and H show paeoniflorin, while C, D, I, and J show albiflorin. E and F display the mass spectrum of the second peak found in the chromatogram of the albiflorin standard measured with method 1. [file BMC-40-e70353-s003.png]

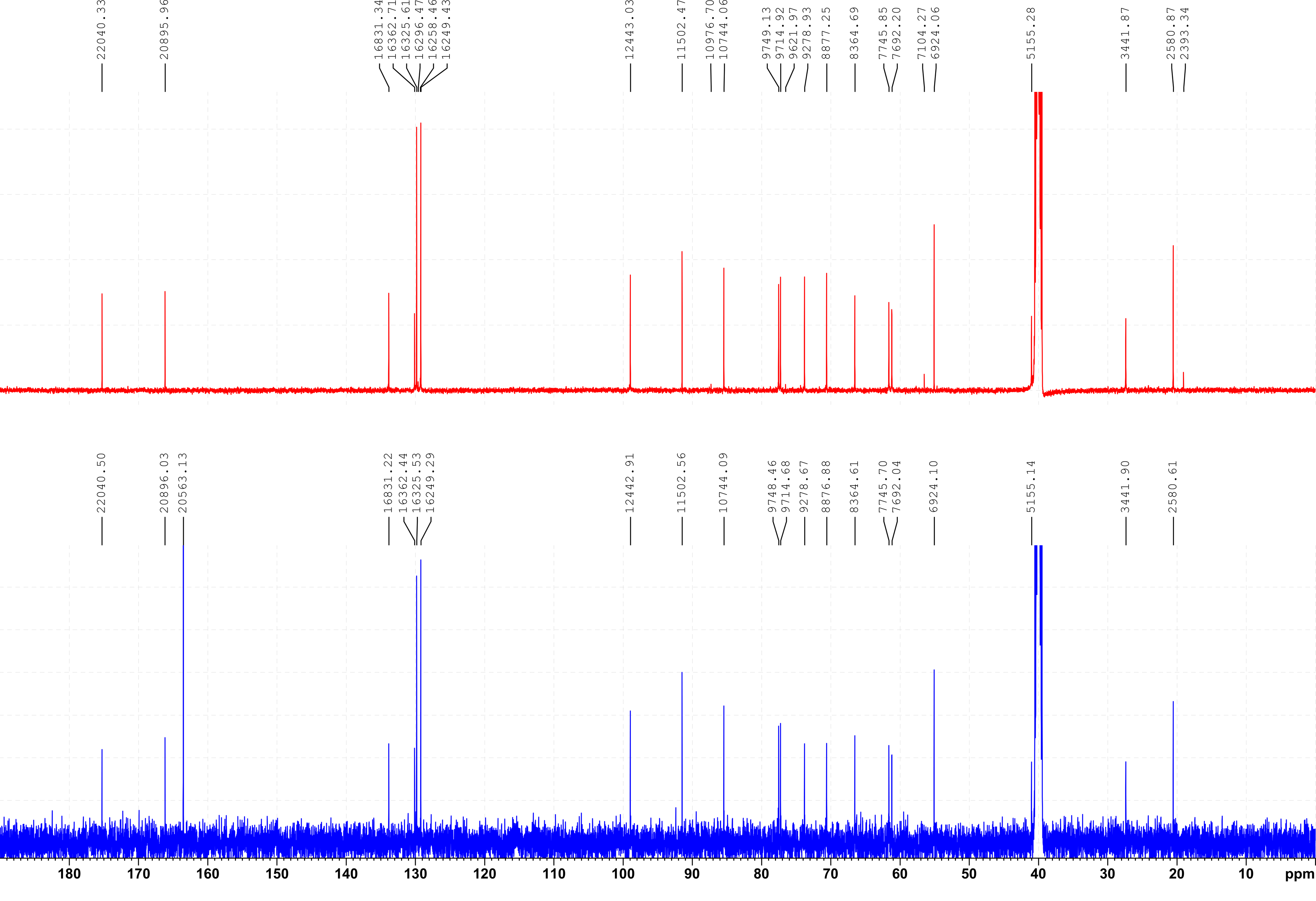

Supplement: Supplementary file 3 — Figure S2: bmc70353‐sup‐0003‐Figure_S2.png. 13C‐NMR spectrum of albiflorin standard before (top, red) and after (bottom, blue) formic acid addition. [file BMC-40-e70353-s007.png]

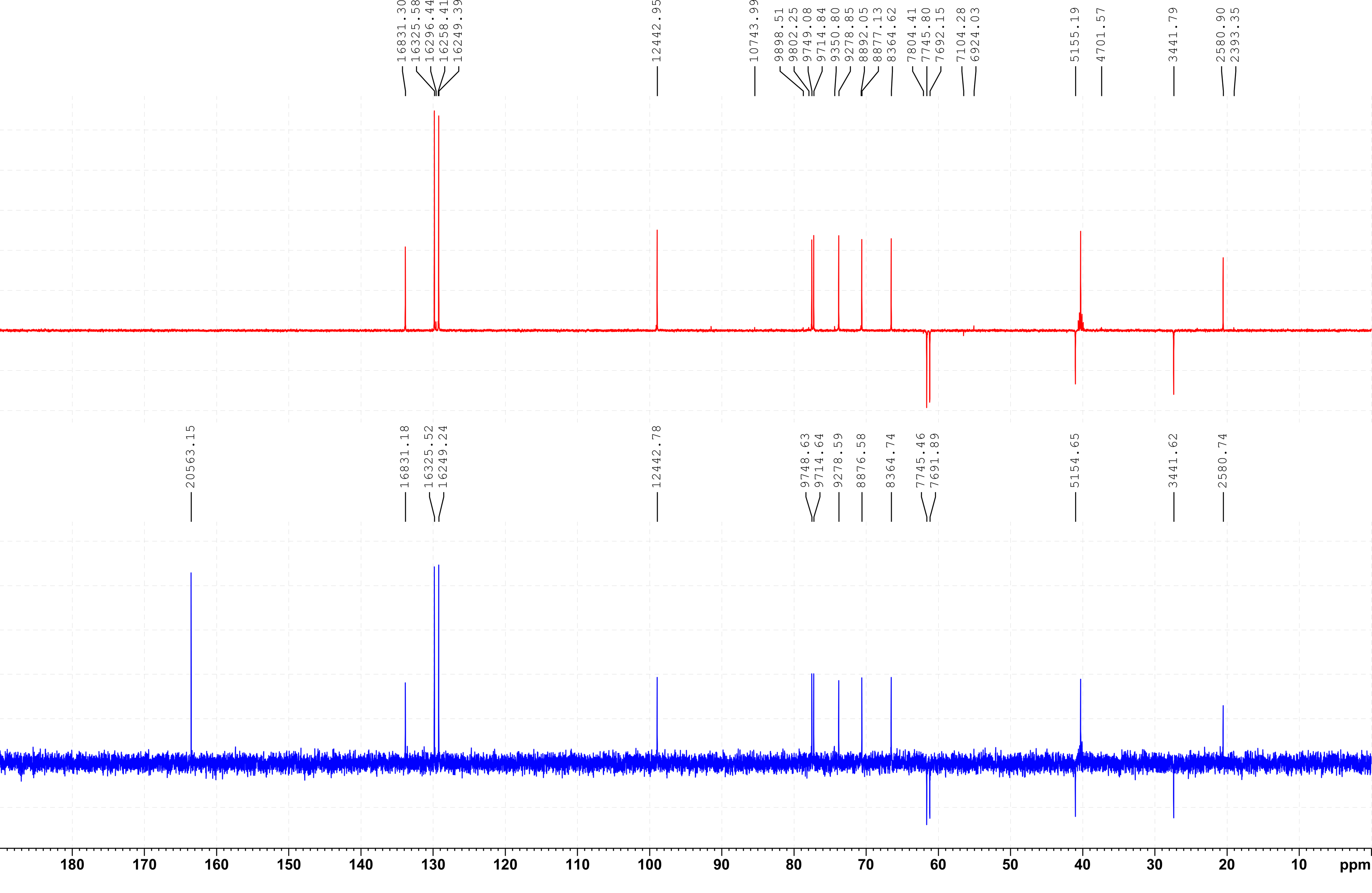

Supplement: Supplementary file 4 — Figure S3: DEPT‐NMR spectrum of albiflorin standard before (top, red) and after (bottom, blue) formic acid addition. [file BMC-40-e70353-s013.png]

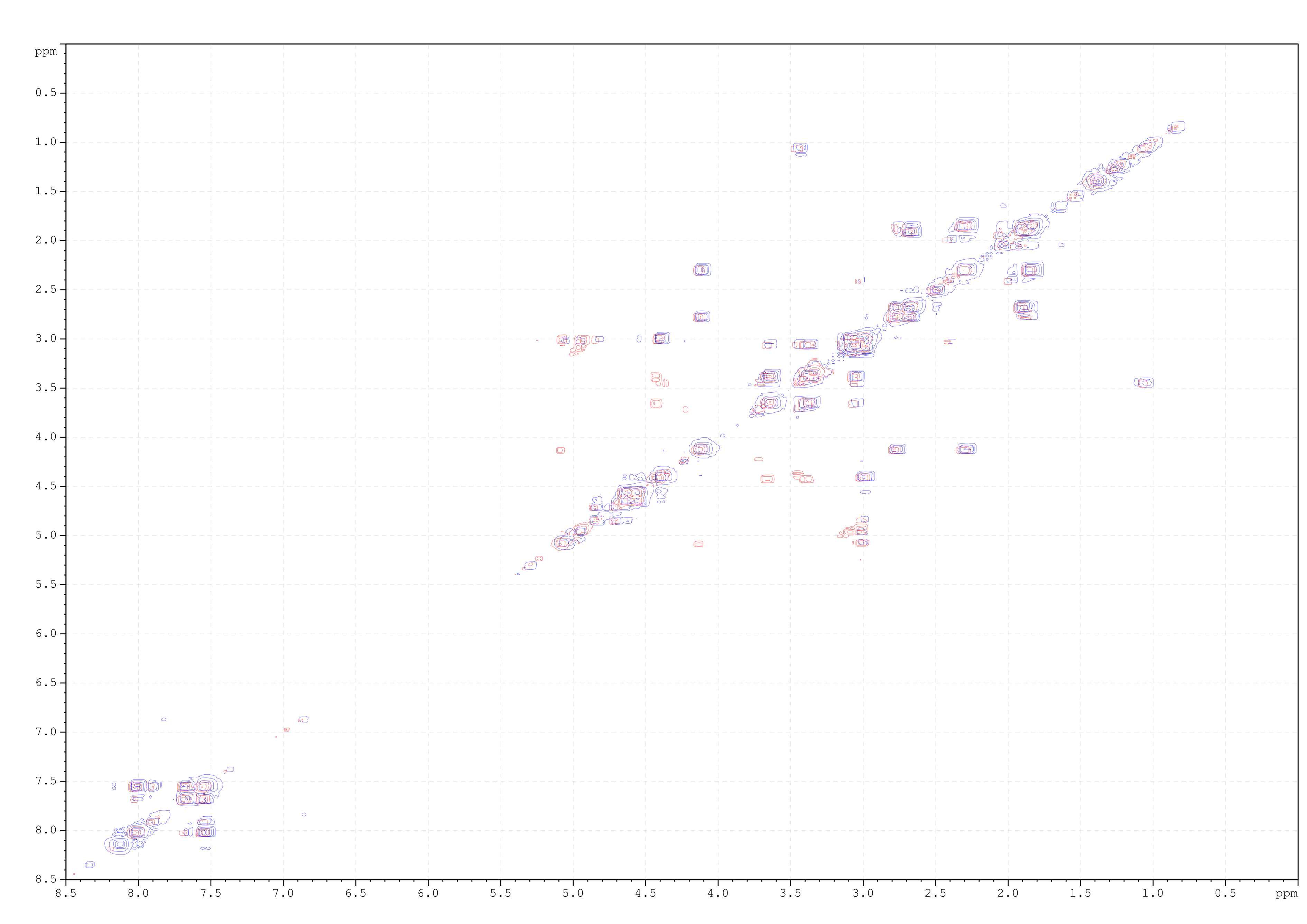

Supplement: Supplementary file 5 — Figure S4: COSY‐NMR spectrum of albiflorin standard before (red) and after (blue) formic acid addition. [file BMC-40-e70353-s014.png]

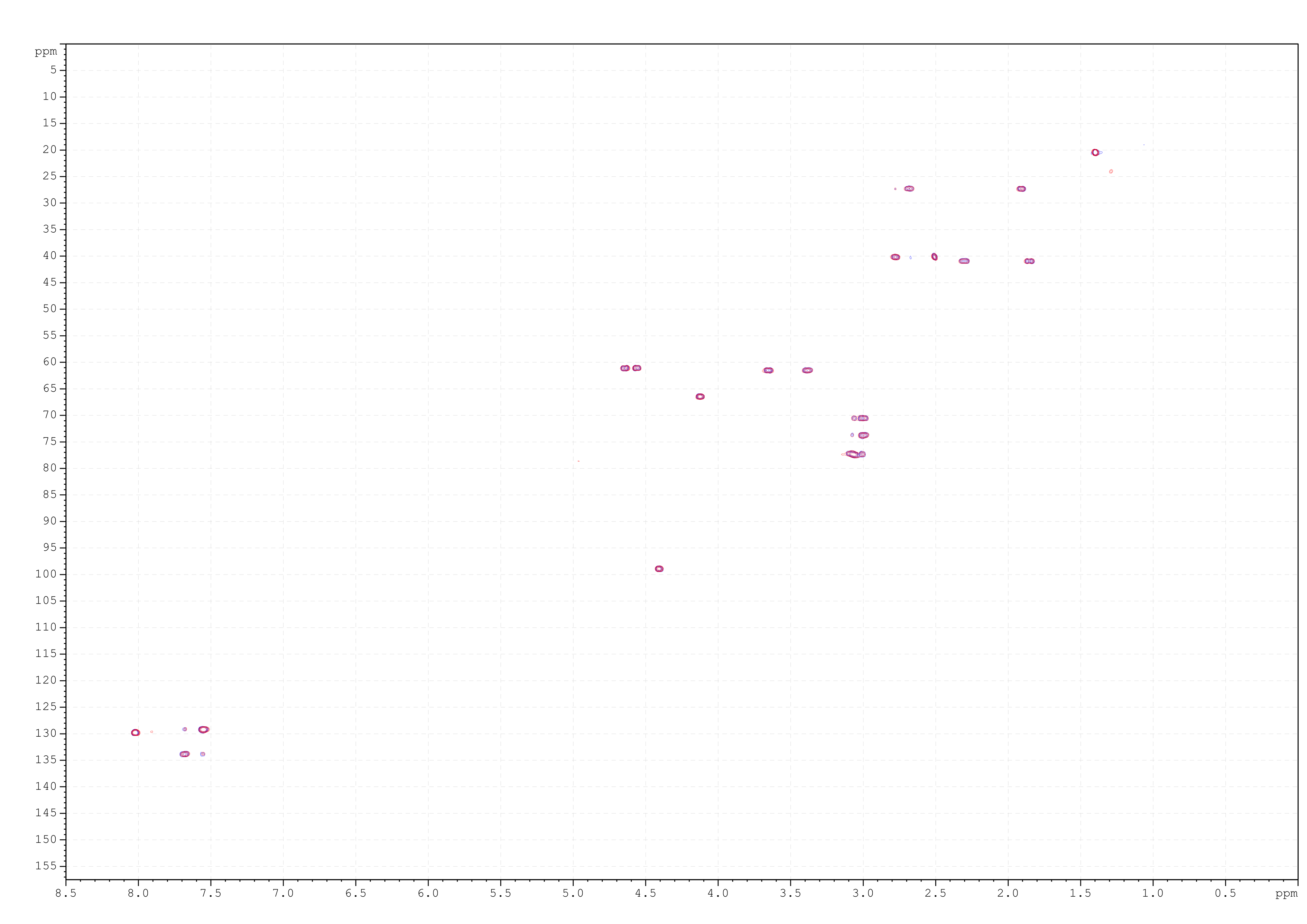

Supplement: Supplementary file 6 — Figure S5: bmc70353‐sup‐0006‐Figure_S5.png. 1H‐13C‐HSQC spectrum of albiflorin standard before (red) and after (blue) formic acid addition. [file BMC-40-e70353-s011.png]

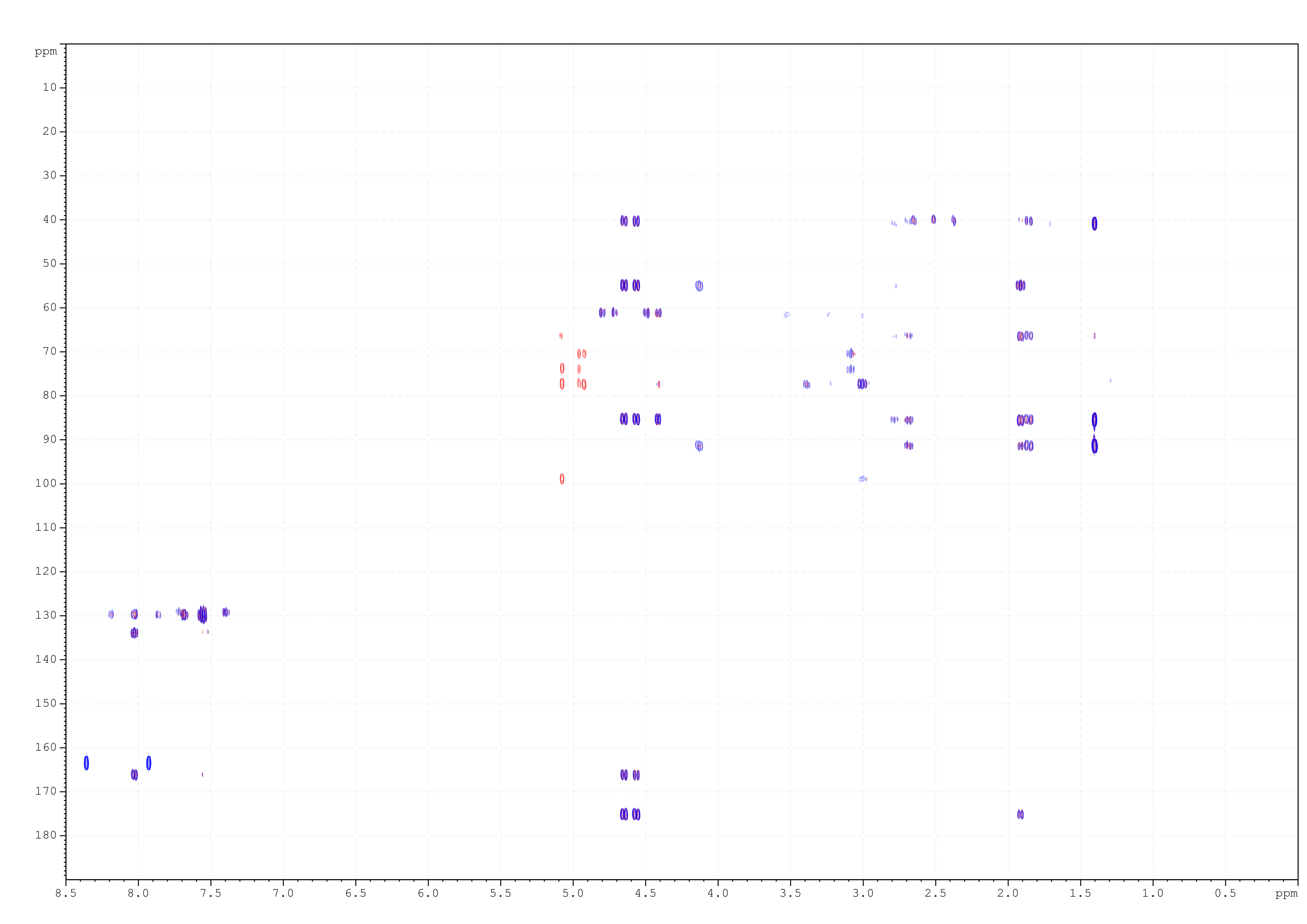

Supplement: Supplementary file 7 — Figure S6: bmc70353‐sup‐0007‐Figure_S6.png. 1H‐13C‐HMBC spectrum of albiflorin standard before (red) and after (blue) formic acid addition. [file BMC-40-e70353-s010.png]

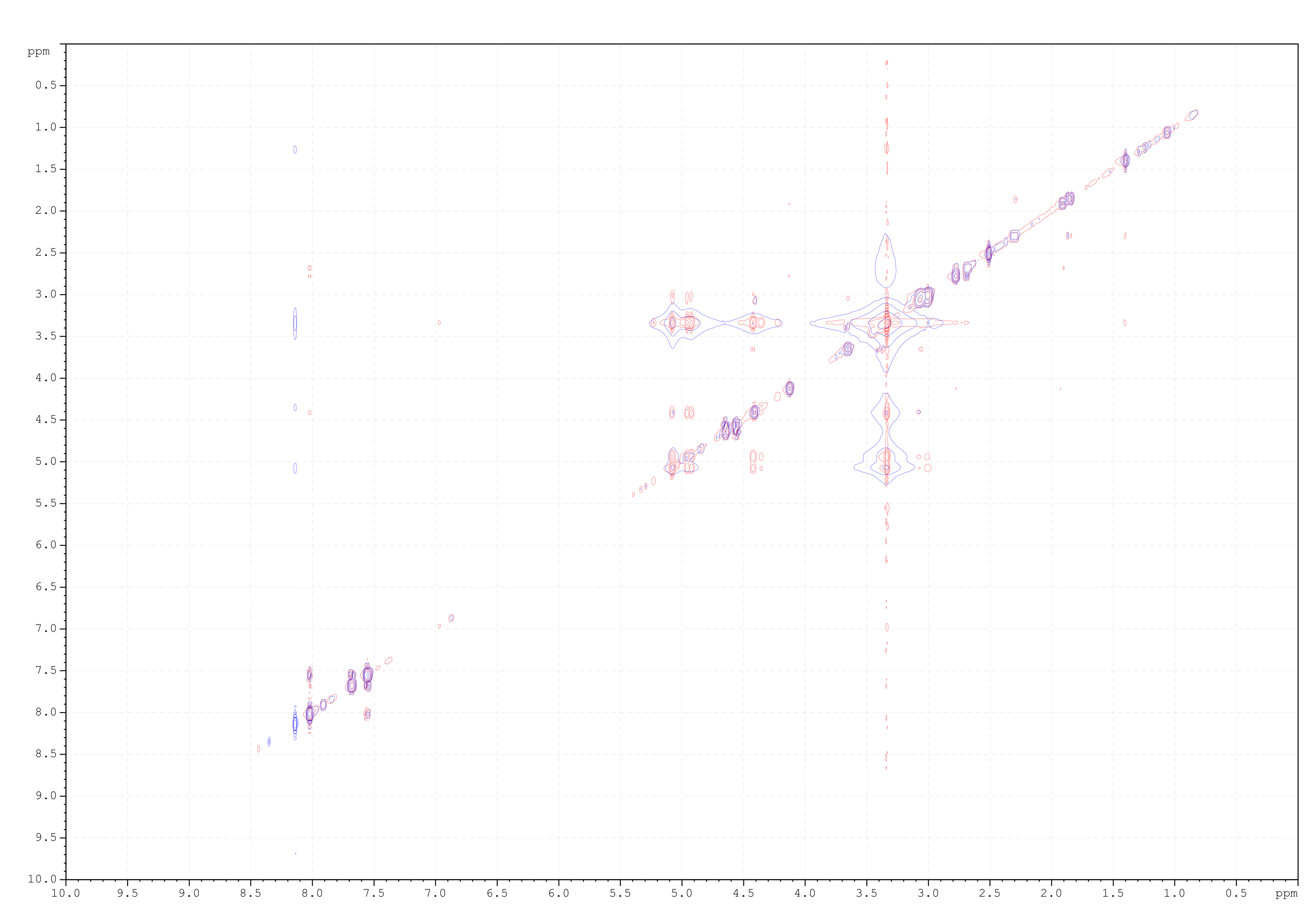

Supplement: Supplementary file 8 — Figure S7: NOESY spectrum of albiflorin standard before (red) and after (blue) formic acid addition. [file BMC-40-e70353-s005.png]

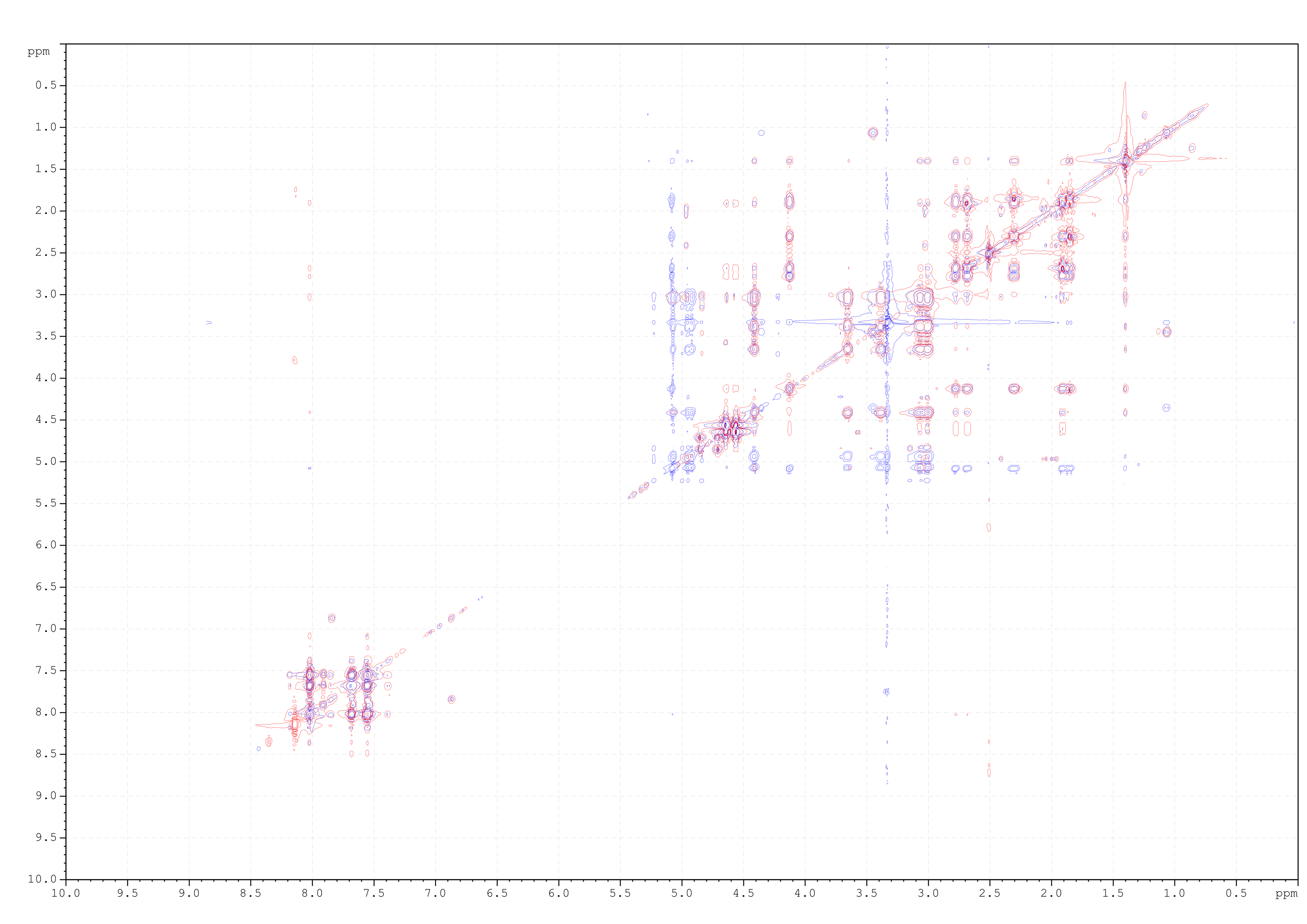

Supplement: Supplementary file 9 — Figure S8: TOCSY spectrum of albiflorin standard before (red) and after (blue) formic acid addition. [file BMC-40-e70353-s009.png]

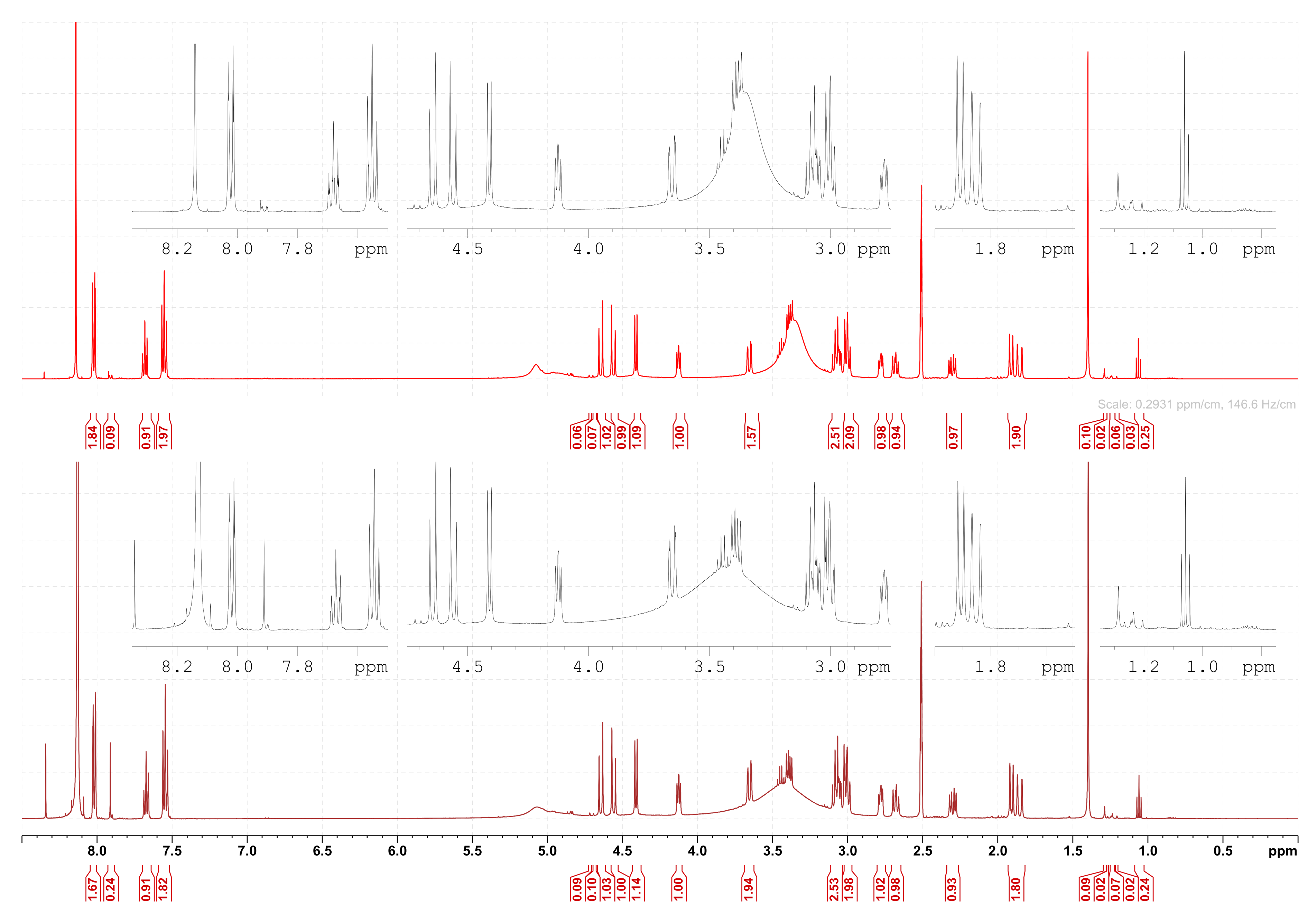

Supplement: Supplementary file 10 — Figure S9: bmc70353‐sup‐0010‐Figure_S9.png. 1H‐NMR spectrum of albiflorin after addition of 1 μL (top, red) and 10 μL (bottom, dark red) of formic acid. [file BMC-40-e70353-s018.png]

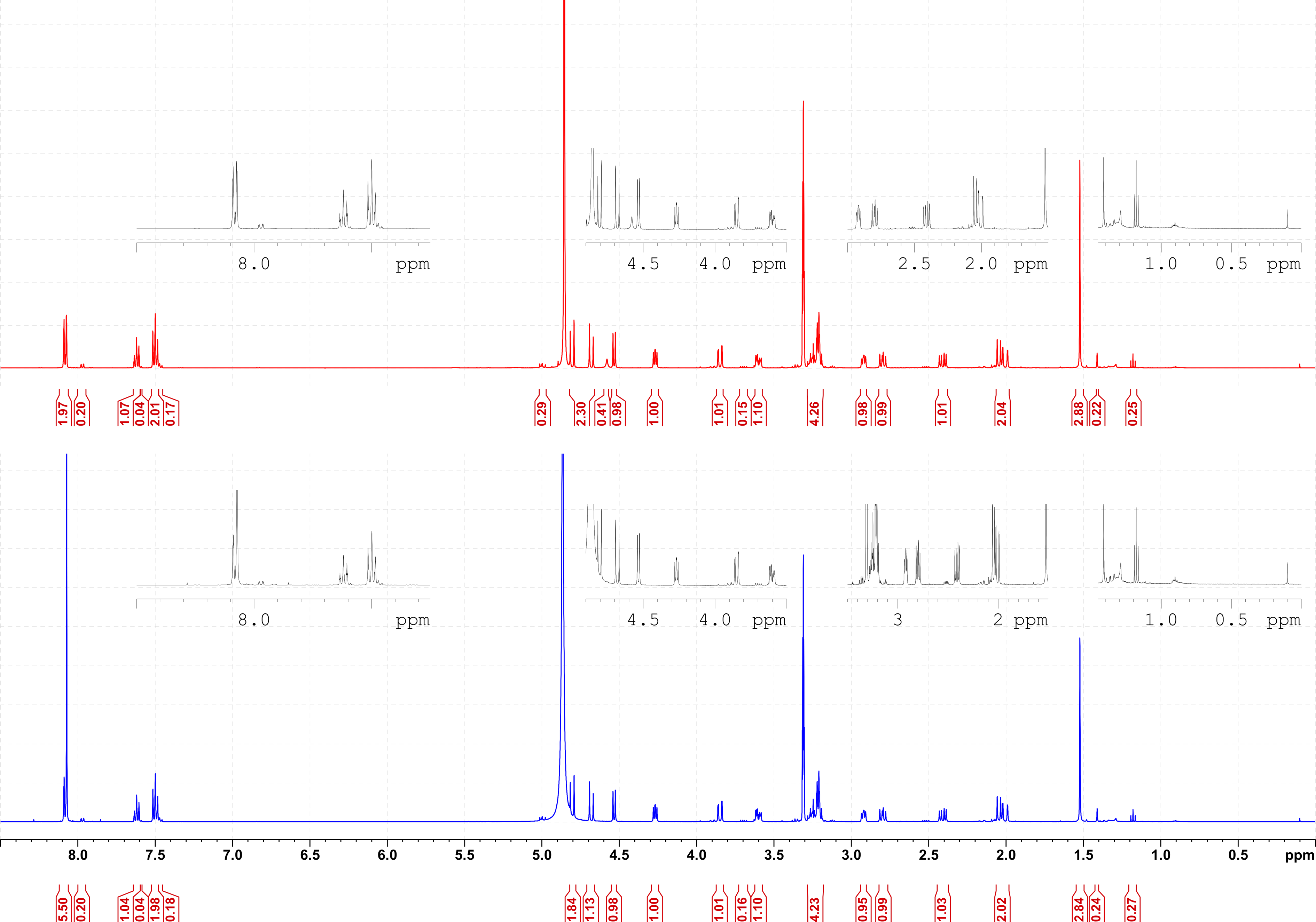

Supplement: Supplementary file 11 — Figure S10: 1H‐NMR spectrum of albiflorin before (top, red) and after (bottom, blue) addition of formic acid. [file BMC-40-e70353-s004.png]

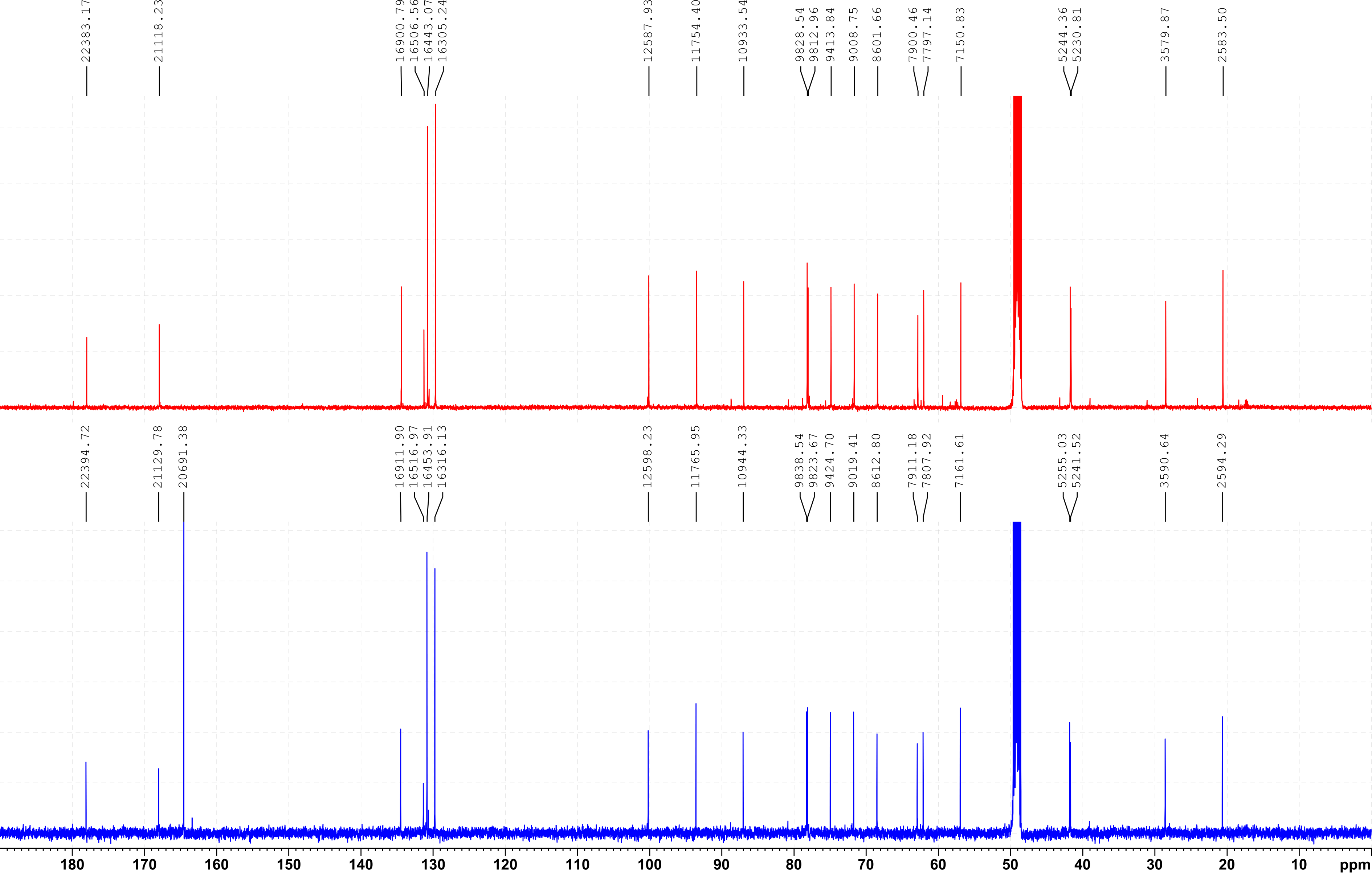

Supplement: Supplementary file 12 — Figure S11: bmc70353‐sup‐0012‐Figure_S11.png. 13C‐NMR spectrum of albiflorin standard before (top, red) and after (bottom, blue) formic acid addition. [file BMC-40-e70353-s017.png]

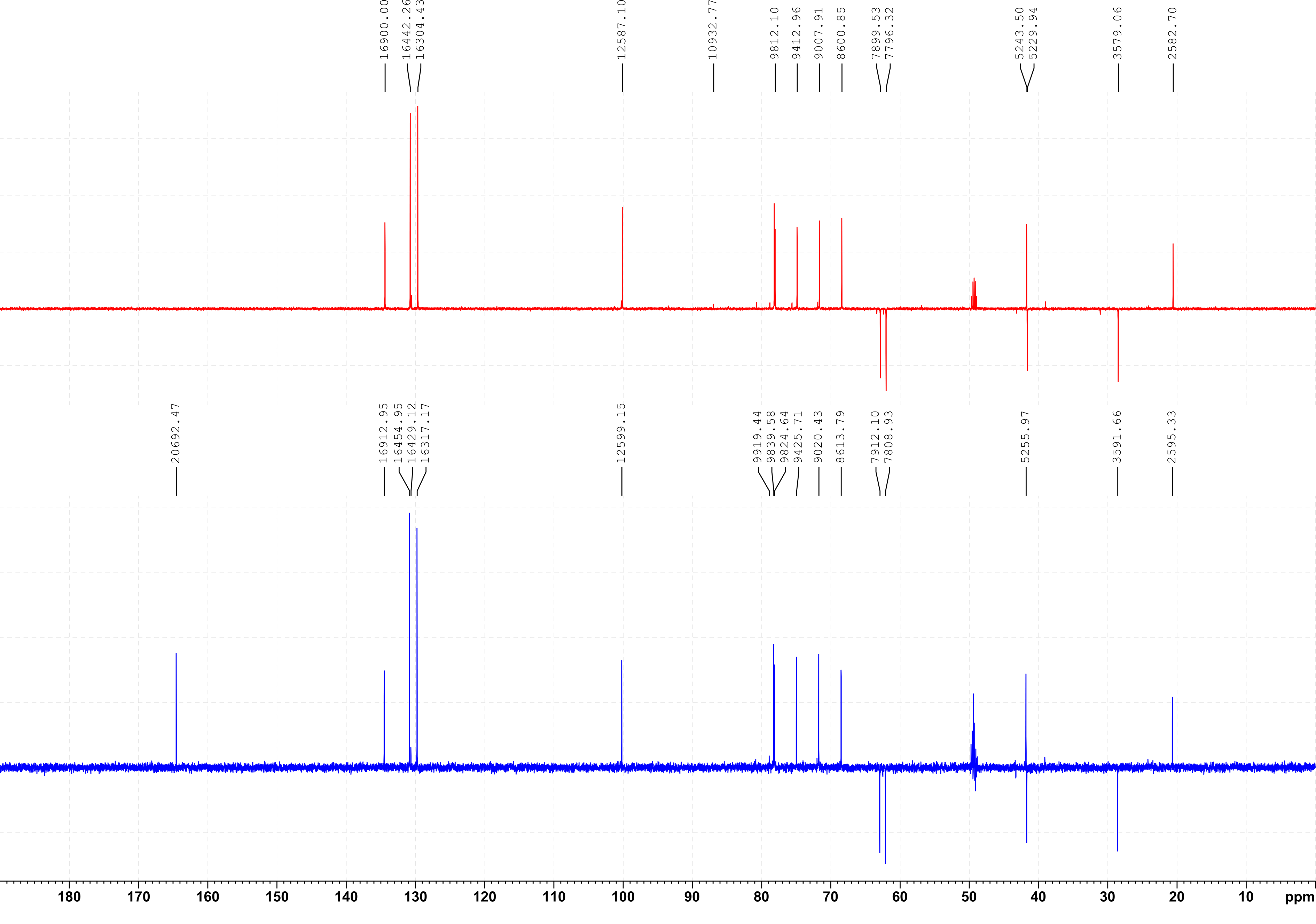

Supplement: Supplementary file 13 — Figure S12: DEPT‐NMR spectrum of albiflorin standard before (top, red) and after (bottom, blue) formic acid addition. [file BMC-40-e70353-s006.png]

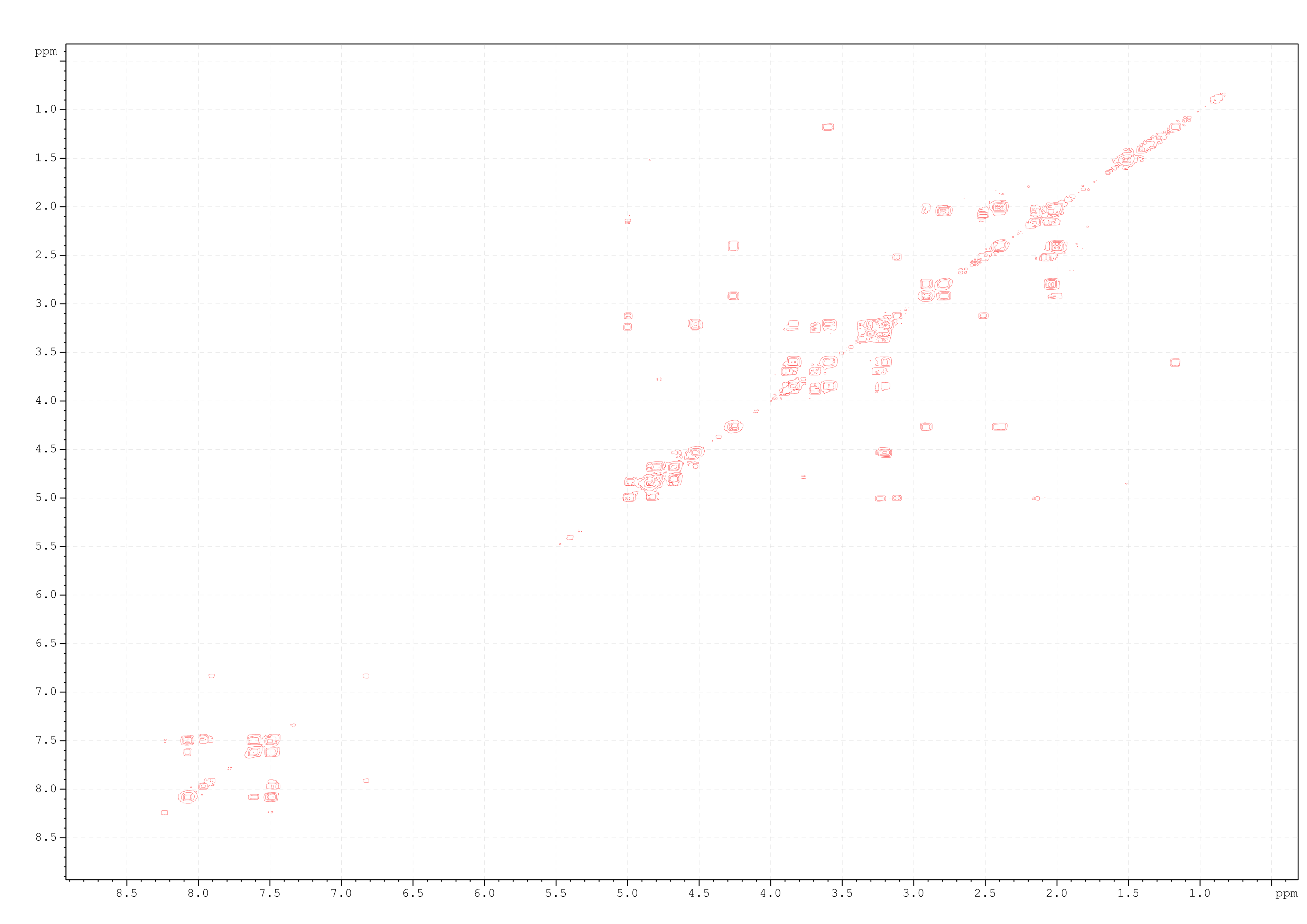

Supplement: Supplementary file 14 — Figure S13: COSY‐NMR spectrum of albiflorin standard after formic acid addition. [file BMC-40-e70353-s019.png]

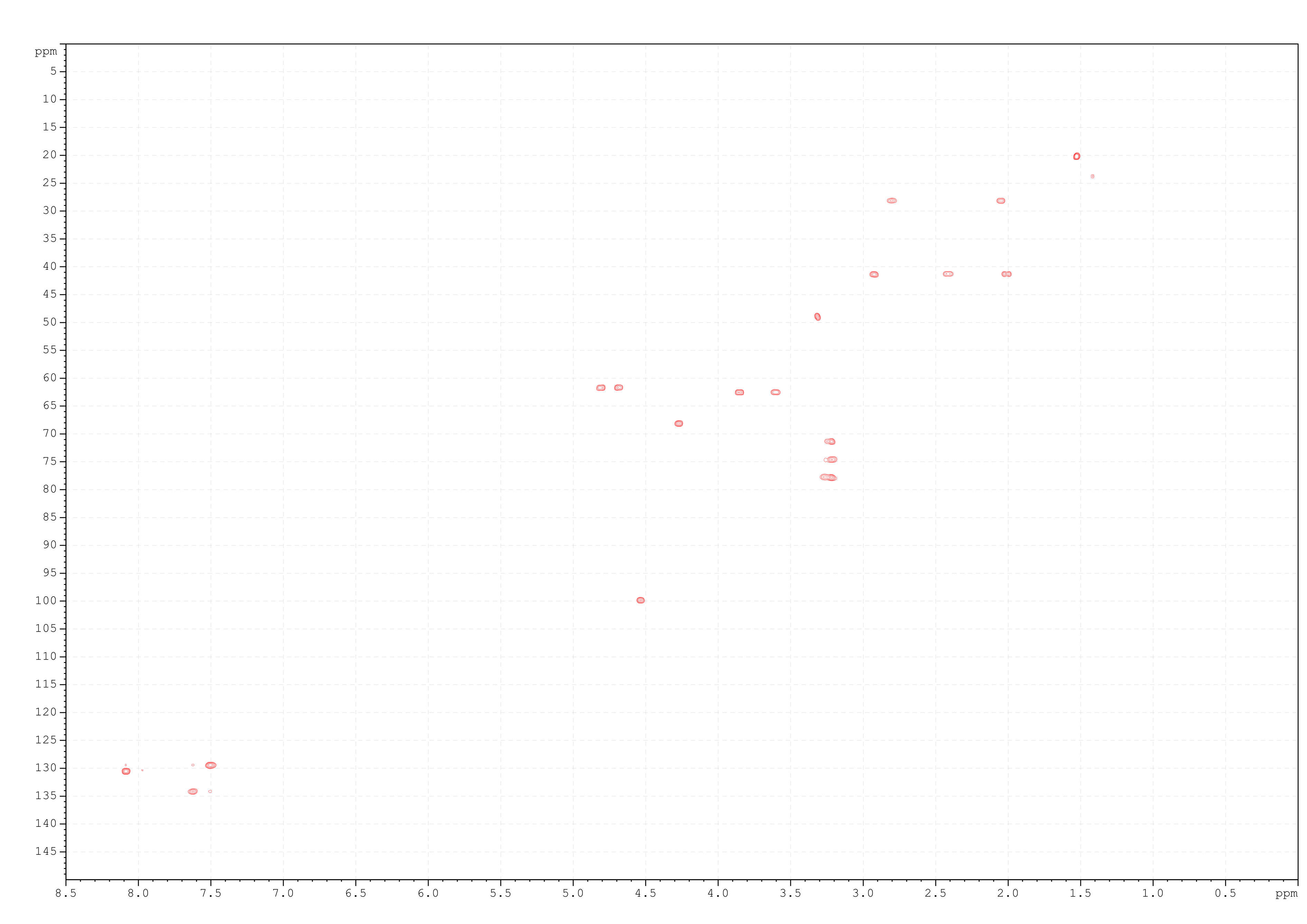

Supplement: Supplementary file 15 — Figure S14: bmc70353‐sup‐0015‐Figure_S14.png. 1H‐13C‐HSQC spectrum of albiflorin standard after formic acid addition. [file BMC-40-e70353-s001.png]

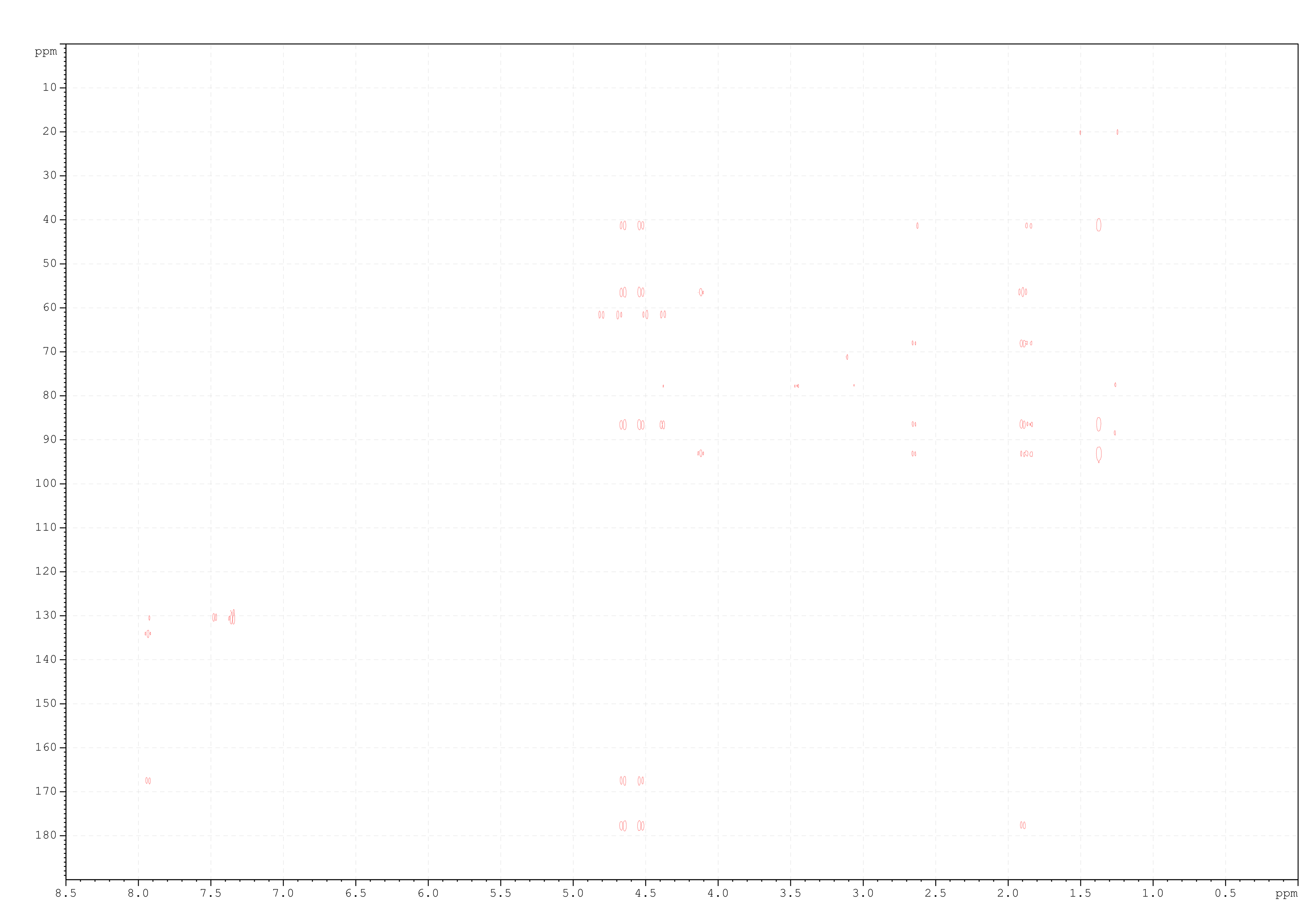

Supplement: Supplementary file 16 — Figure S15: 1H‐13C‐HMBC spectrum of albiflorin standard after formic acid addition. [file BMC-40-e70353-s016.png]

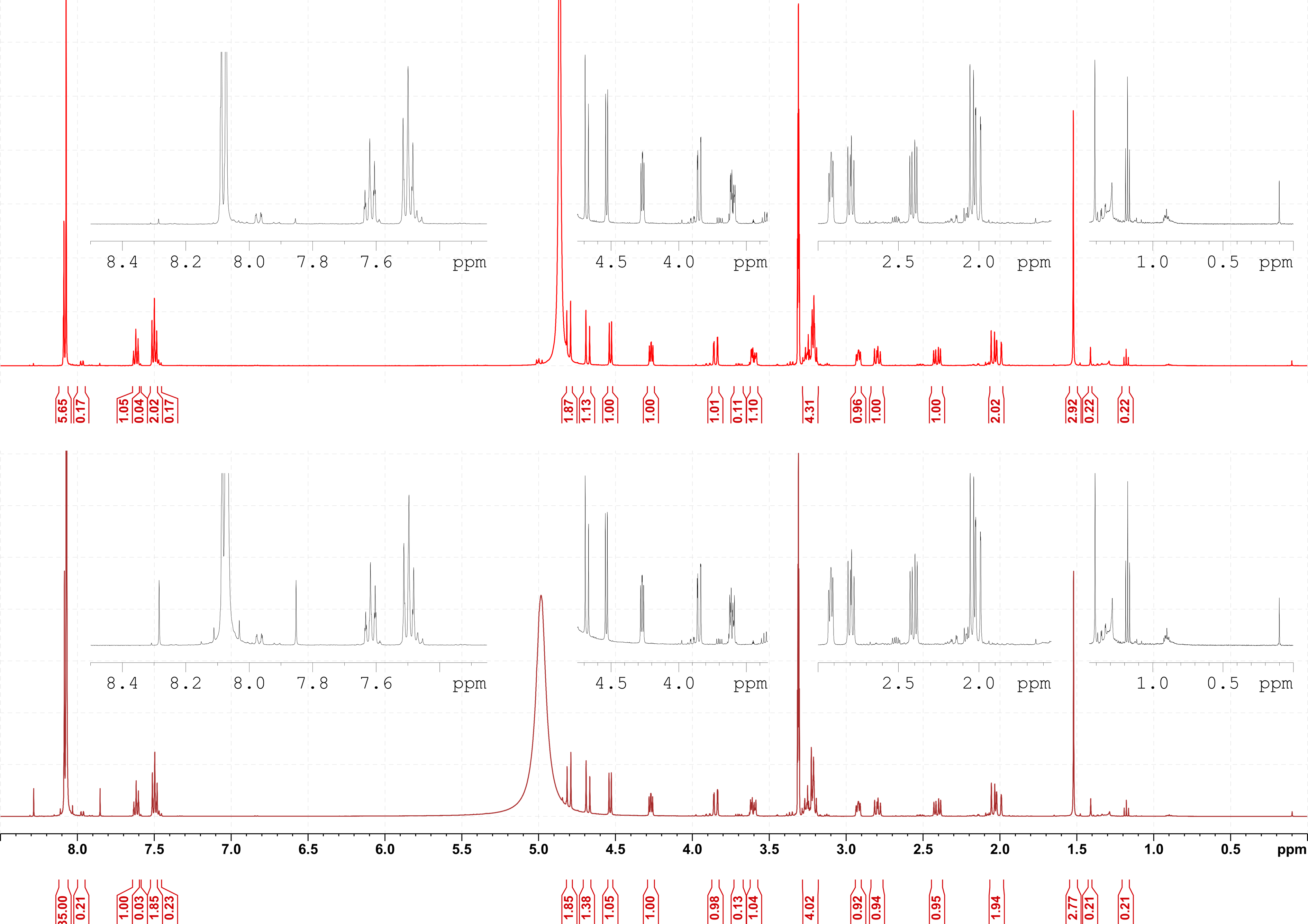

Supplement: Supplementary file 17 — Figure S16: bmc70353‐sup‐0017‐Figure_S16.png. 1H‐NMR spectrum of albiflorin after addition of 1 μL (top, red) and 10 μL (bottom, dark red) of formic acid. [file BMC-40-e70353-s008.png]

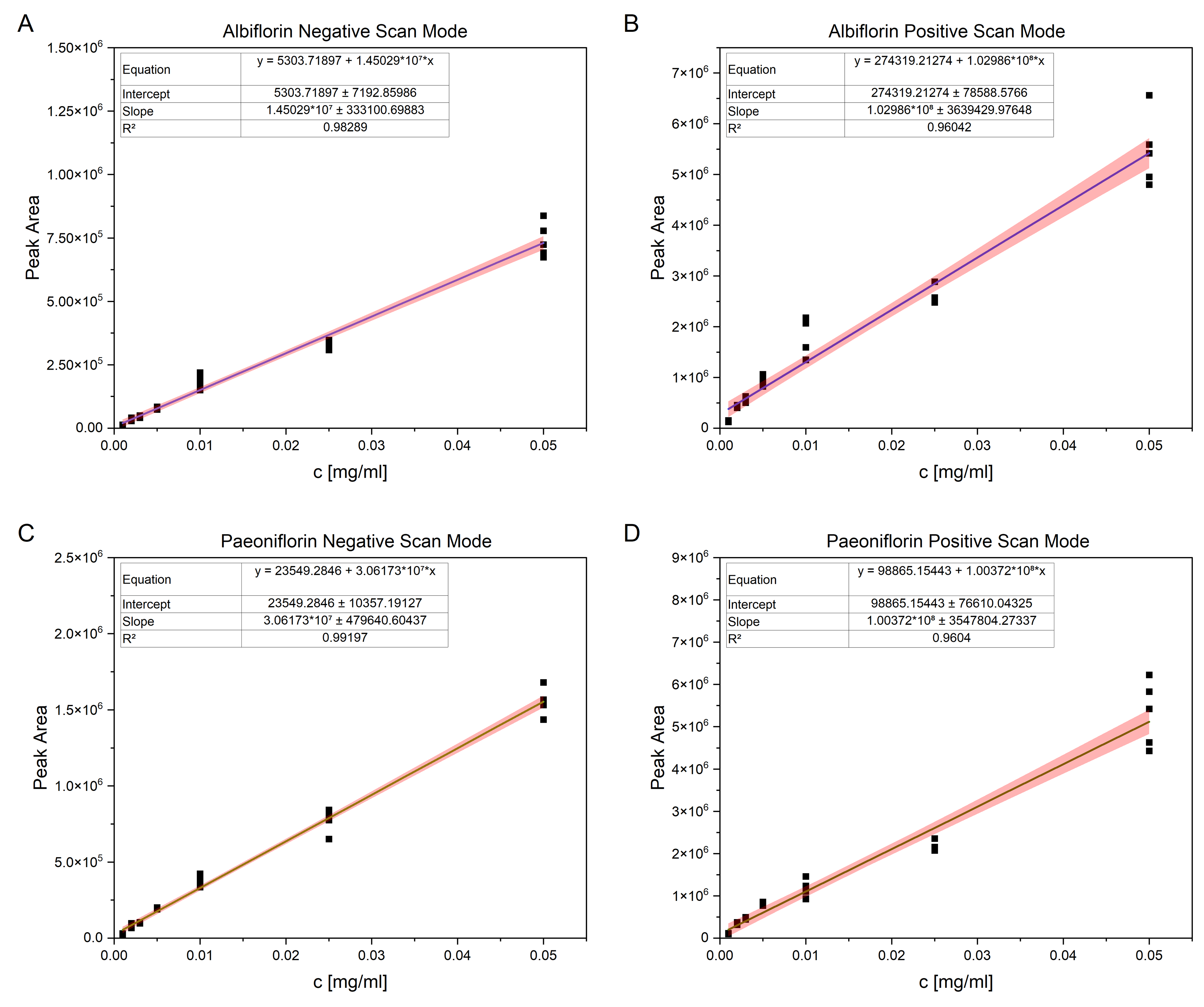

Supplement: Supplementary file 18 — Figure S17: Calibration curves of albiflorin (A, B) and paeoniflorin (C, D) in negative and positive scan modes. The fitted calibration equations, coefficients of determination (R 2) and 95% confidence bands are shown. [file BMC-40-e70353-s012.png]

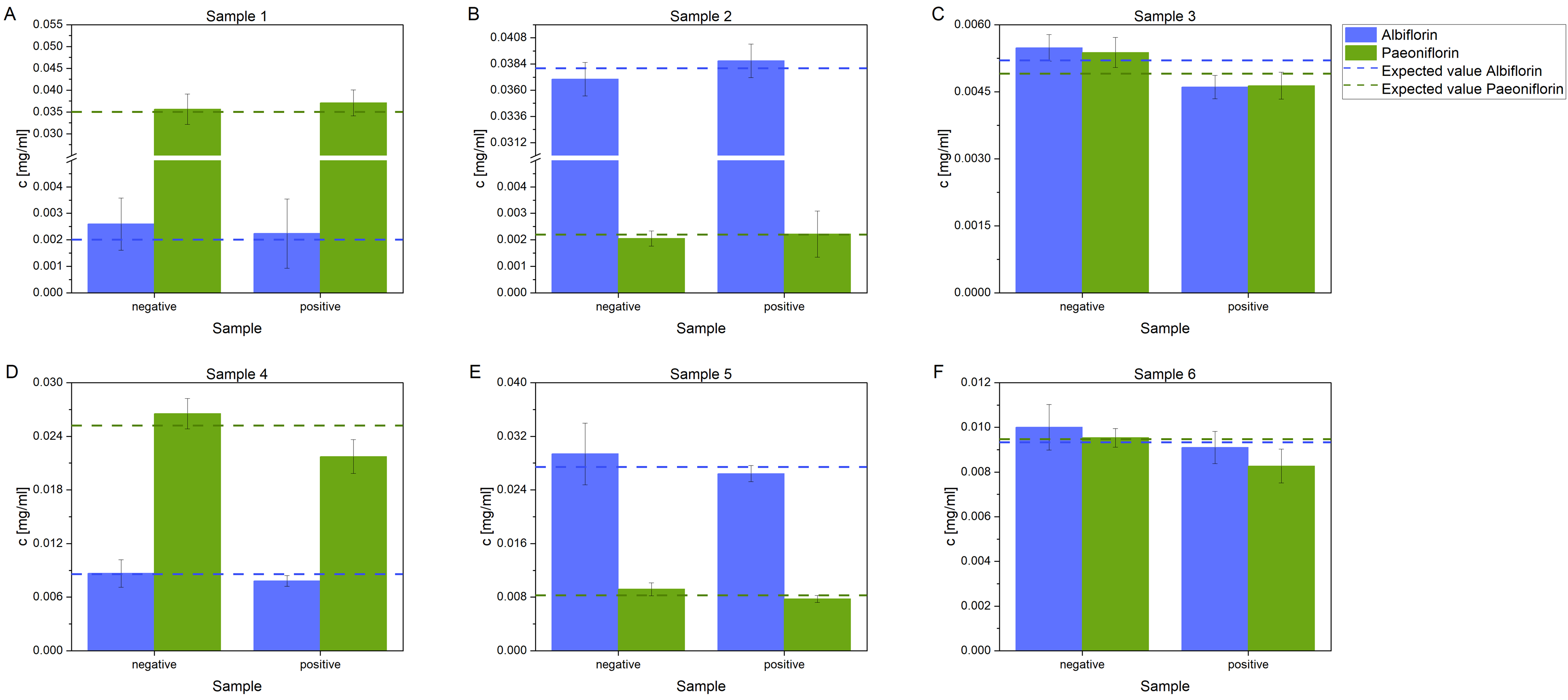

Supplement: Supplementary file 19 — Figure S18: Measured concentrations of albiflorin (blue) and paeoniflorin (green) in the six validation samples (A–F) in negative and positive scan modes. Dashed lines represent the expected (nominal) values for albiflorin and paeoniflorin and error bars indicate standard deviations of replicate measurements. [file BMC-40-e70353-s015.png]
